# Alternation between taxonomically divergent hosts is not the major determinant of flavivirus evolution

**DOI:** 10.1093/ve/veab040

**Published:** 2021-04-21

**Authors:** Chiara Pontremoli, Diego Forni, Mario Clerici, Rachele Cagliani, Manuela Sironi

**Affiliations:** Scientific Institute IRCCS E. MEDEA, Bioinformatics, Bosisio Parini 23842, Italy; Scientific Institute IRCCS E. MEDEA, Bioinformatics, Bosisio Parini 23842, Italy; Department of Physiopathology and Transplantation, University of Milan, Milan 20122, Italy; Don C. Gnocchi Foundation ONLUS, IRCCS, Milan 20121, Italy; Scientific Institute IRCCS E. MEDEA, Bioinformatics, Bosisio Parini 23842, Italy; Scientific Institute IRCCS E. MEDEA, Bioinformatics, Bosisio Parini 23842, Italy

**Keywords:** Flavivirus evolution, Dating analysis, Episodic positive selection, Tick-borne flavivirus, Mosquito-borne flavivirus, Hosts alternation

## Abstract

Flaviviruses display diverse epidemiological and ecological features. Tick-borne and mosquito-borne flaviviruses (TBFV and MBFV, respectively) are important human pathogens that alternate replication in invertebrate vectors and vertebrate hosts. The *Flavivirus* genus also includes insect-specific viruses (ISFVs) and viruses with unknown invertebrate hosts. It is generally accepted that viruses that alternate between taxonomically different hosts evolve slowly and that the evolution of MBFVs and TBFVs is dominated by strong constraints, with limited episodes of positive selection. We exploited the availability of flavivirus genomes to test these hypotheses and to compare their rates and patterns of evolution. We estimated the substitution rates of CFAV and CxFV (two ISFVs) and, by taking into account the time-frame of measurement, compared them with those of other flaviviruses. Results indicated that CFAV and CxFV display relatively different substitution rates. However, these data, together with estimates for single-host members of the *Flaviviridae* family, indicated that MBFVs do not display relatively slower evolution. Conversely, TBFVs displayed some of lowest substitution rates among flaviviruses. Analysis of selective patterns over longer evolutionary time-frames confirmed that MBFVs evolve under strong purifying selection. Interestingly, TBFVs and ISFVs did not show extremely different levels of constraint, although TBFVs alternate among hosts, whereas ISFVs do not. Additional results showed that episodic positive selection drove the evolution of MBFVs, despite their high constraint. Positive selection was also detected on two branches of the TBFVs phylogeny that define the seabird clade. Thus, positive selection was much more common during the evolution of arthropod-borne flaviviruses than previously thought. Overall, our data indicate that flavivirus evolutionary patterns are complex and most likely determined by multiple factors, not limited to the alternation between taxonomically divergent hosts. The frequency of both positive and purifying selection, especially in MBFVs, suggests that a minority of sites in the viral polyprotein experience weak constraint and can evolve to generate new viral phenotypes and possibly promote adaptation to new hosts.

## 1. Introduction

Flaviviruses (family *Flaviviridae*, genus *Flavivirus*) are single-stranded, positive sense RNA viruses responsible for a number of emerging and re-emerging diseases. Some of these human pathogens, such as yellow fever virus, dengue virus (DENV), West Nile virus (WNV), and Zika virus (ZIKV) are the causative agents of large-scale epidemics that result in millions of human infections every year (Gould et al. 2017). Others, including Japanese encephalitis virus, St Louis encephalitis virus (SLEV), Usutu virus, Spondweni virus, and tick-borne encephalitis virus (TBEV) are responsible for more localized outbreaks. All these viruses are transmitted to humans, as well as to other domestic and wild animals, by arthropod vectors.

**Figure 1.**
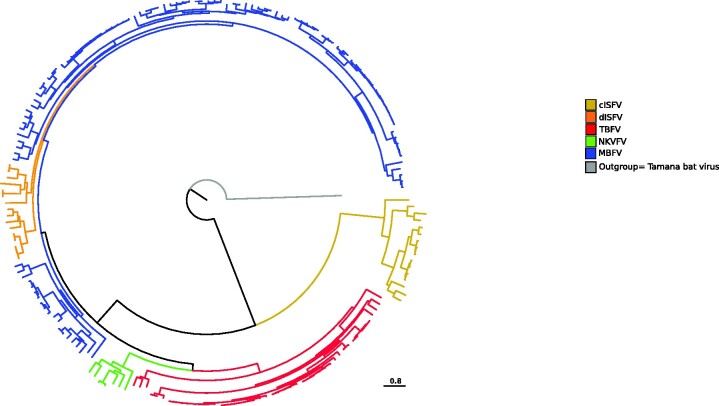
Flavivirus phylogenetic tree. Maximum likelihood phylogeny of representative viruses belonging to the MBFV, TBFV, NKVFV, cISFV, and dISFV groups. Tamana bat virus was used as the outgroup.

Most vertebrate-infecting flaviviruses can be divided into three large groups. Tick-borne flaviviruses (TBFV) and mosquito-borne flaviviruses (MBFV) have variable host ranges and can infect different vertebrate hosts, including birds and mammals (Pandit et al. 2018). However, vertebrate-specific flaviviruses also exist, which seem to have no arthropod vector and can be transmitted horizontally among mammals. These viruses are commonly referred to as no known vector flaviviruses (NKVFV) and can be further divided into bat- and rodent-associated NKVFVs (Blitvich and Firth 2017). These different transmission modes are well reflected in the phylogenetic relationships among flaviviruses (Fig. 1). The only inconsistency involves three viruses (Entebbe bat virus, Yokose virus, and Sokoluk virus), which have no known vector but display close genetic similarity to MBFVs. It is presently unclear whether these viruses have lost the ability to infect mosquitoes or if their vectors are still unknown (Blitvich and Firth 2017).

The *Flavivirus* genus also includes a growing number of insect-specific viruses (ISFVs) (Fig. 1). The so-called classical ISFVs (cISFVs) are phylogenetically distinct from all other flaviviruses and naturally infect mosquitoes, whereas another ISFV group (dual host-affiliated ISFVs, dISFVs) clusters with MBFVs (Fig. 1). dISFVs might thus infect unidentified vertebrate hosts or they may have recently lost the ability to replicate in vertebrates (Blitvich and Firth 2015). This latter possibility seems to be more likely, as *in vitro* experiments indicated that dISFVs cannot replicate in a number of vertebrate cell lines (Blitvich and Firth 2015).

The differences in host ranges and transmission cycles suggest that flaviviruses are subject to diverse selective pressures and evolutionary trajectories. A long-standing hypothesis holds that viruses that naturally alternate between different hosts evolve less rapidly than those that specialize in a single host, as optimization for replication in one host may reduce fitness for infection of the other (Weaver et al. 1999; Jenkins et al. 2002; Jenkins et al. 2002; Holmes 2003; Greene et al. 2005; Coffey et al. 2008, 2013; Vasilakis et al. 2009). This may be especially true for viruses that infect both vertebrates and invertebrates, as these hosts display fundamentally different tissue organization and antiviral responses (Coffey et al. 2013). This hypothesis, however, was shown to be only partially true in the case of WNV and SLEV, two MBFVs (Ciota et al. 2007, 2008, 2009; Deardorff et al. 2011).

Several studies have shown that the intra- and inter-host evolution of MBFVs and TBFVs is dominated by strong constraints, which seems to differ among hosts (Coffey et al. 2013; Sessions et al. 2015; Grubaugh et al. 2015, 2016a; Nelson et al. 2018). The notion that strong functional constraint drives the evolution of MBFVs and TBFVs is paralleled by the observation that positive (diversifying) selection is less common in vector-borne viruses compared with viruses transmitted by other routes (Woelk and Holmes 2002).

An additional layer of complexity in the evolution of vector-borne flaviviruses lies in the severe bottlenecks that occur during horizontal transmission from vertebrates to vectors and within the vectors, as a result of anatomical barriers (Ciota et al. 2012; Forrester et al. 2012; Coffey et al. 2013; Sim et al. 2015; Gutiérrez et al. 2015; Grubaugh et al. 2016a,b; Lequime et al. 2016; Grubaugh and Ebel 2016). Bottlenecks are non-selective reductions of the effective viral population size, as well as of genetic diversity. Despite these events, MBFVs and TBFVs recover diversity through population expansions in different hosts (Coffey et al. 2013; Grubaugh et al. 2016b; Grubaugh and Ebel 2016). Moreover, genetic diversification is promoted by antiviral RNAi (RNA interference) responses in vectors (Brackney et al. 2009; Schnettler et al. 2014; Brackney et al. 2015; Grubaugh et al. 2016a,b). RNAi, a major antiviral mechanism in insects and non-insect arthropods, results in the sequence-specific degradation of viral RNA (Pijlman 2014; Olson and Blair 2015; Leggewie and Schnettler 2018). Thus, RNAi tends to select for rare variants, either synonymous or non-synonymous, that avoid sequence complementarity (Brackney et al. 2009; Schnettler et al. 2014; Brackney et al. 2015; Lambrechts and Lequime 2016; Grubaugh et al. 2016a,b).

Because they infect taxonomically diverse hosts and display distinct transmission routes, flaviviruses are expected to face extremely different pressures and, consequently, to display distinctive evolutionary trajectories. We exploited the availability of fully sequenced genomes of MBFVs, TBFVs, NKVFVs, cISFVs, and dISFVs to compare their rates and patterns of evolution.

## 2. Materials and methods

### 2.1 Sequence selection, alignments, and phylogenies

Sequence data for cell fusing agent virus (CFAV) and Culex flavivirus (CxFV) strains with known collection date were retrieved from the NCBI database (http://www.ncbi.nlm.nih.gov/) (Supplementary Table S1).

Likewise, MBFV (*n* = 110), TBFV (*n* = 46), NKVFV (*n* = 7), cISFV (*n* = 16), and dISFV (*n* = 13) coding sequences for the whole polyprotein were obtained from the NCBI (http://www.ncbi.nlm.nih.gov/) and ViPR (Virus Pathogen Resource, https://www.viprbrc.org) databases. For each group, strains were chosen to be representative of all species with complete or nearly complete genomes present in the ICTV flavivirus phylogenetic tree (https://talk.ictvonline.org/ictv-reports/ictv_online_report/positive-sense-rna-viruses/w/flaviviridae). dISFV strains were selected as in Blitvich and co-workers review (Blitvich and Firth 2015). A list of accession numbers is reported in Supplementary Table S2 and a representation of the flavivirus phylogenetic tree is shown in Fig. 1.

MAFFT (Katoh and Standley 2013) was used to generate multiple sequence alignments and GUIDANCE2 (Sela et al. 2015) for filtering unreliably aligned codons (confidence score < 0.90) (Privman et al. 2012).

Alignments were screened for the presence of recombination using GARD (Kosakovsky Pond et al. 2006). GARD uses phylogenetic incongruence among segments in the alignment and the statistical significance of putative breakpoints is evaluated through Kishino-Hasegawa (HK) tests. No significant breakpoints (*p* < 0.01) were detected.

To evaluate the level of substitution saturation at the third codon position, the Xia's index implemented in DAMBE (Xia et al. 2003; Xia 2013) was applied (Supplementary Table S3). This test compares an entropy-based index of saturation (I_ss_) with a critical value (I_ss.c_). If I_ss_ is significantly lower than I_ss.c_, sequences have not experienced substitution saturation.

Finally, phylogenetic trees were reconstructed using the phyML program (version 3.0) with a maximum-likelihood approach, a General Time Reversible model plus gamma-distributed rates and 4 substitution rate categories(Guindon et al. 2009).

### 2.2 Substitution rate estimates

Sequence data for the coding sequence of the envelope (E) proteins of CFAV (*n* = 49) and CxFV with known collection date were analyzed (Supplementary Table S1). For CxFV, the two major clades (Asia/USA genotype, *n* = 77, and Africa/Caribbean/Latin America genotype, *n* = 47) (Bittar et al. 2016; Miranda et al. 2019) were separately analyzed. For CFAV, forty-eight coding sequences of the ns5 and ns3 proteins were retrieved.

To evaluate whether the phylogenies carried sufficient temporal signal, the correlation coefficients (*r*) of regressions of root-to-tip genetic distances against sequence sampling years were calculated (Murray et al. 2016). A method that minimizes the residual mean squares of the models was applied and *z*s were calculated by performing 1,000 permutations of sampling dates (Duchene et al. 2015; Murray et al. 2016). In the case of CFAV E coding sequence, one clear outlier (AB488425) was observed and removed from further analyses. The reason(s) why this sequence, which was the shortest in the dataset, behaved as an outlier are unknown.

Phylogenetic reconstruction was performed using a Bayesian approach implemented in the Bayesian Evolutionary Analysis by Sampling Trees (BEAST, v.1.10.4) software (Suchard et al. 2018). A Coalescent Exponential tree prior and a relaxed log normal clock were used in all analyses. We performed two different runs, one hundred million iterations each, and sampled every 10,000 steps after a 10% burn-in. Runs were combined after checking for convergence and for heaving effective sampling sizes >100. A maximum clade credibility tree was generated using TreeAnnotator (Bouckaert et al. 2014) and visualized with FigTree (http://tree.bio.ed.ac.uk/) (Supplementary Fig. S1).

The estimated rates of viral evolution scale negatively with the time-frame over which they are measured (Aiewsakun and Katzourakis 2016; Simmonds et al. 2019). Because of this time-dependent rate phenomenon, and to take into account the time span of sequence sampling, estimates for members of the *Flaviviridae* family were retrieved from previous works (Subbotina and Loktev 2012; McMullen et al. 2013; Karan et al. 2014; Di Giallonardo et al. 2015; Faria et al. 2016; Simmonds et al. 2019; Clark et al. 2020) and compared with the CFAV and to the CxFV substitution rates (Supplementary Table S4). Log10-transformed rates were then plotted as a function of log10-transformed timescales.

### 2.3 Positive selection analysis

To obtain an estimate of the selective constraint, the dN/dS parameter was calculated using the single-likelihood ancestor counting (SLAC) method (Kosakovsky Pond and Frost 2005) for all viral proteins (the 2k peptide, which is only 23-bp long, was merged with the ns4a protein) (Supplementary Table S5).

To investigate whether episodic positive selection acted on the internal branches of the five flavivirus groups, the adaptive Branch-Site Random Effects Likelihood method (aBSREL) (Smith et al. 2015) was used. This method applies sequential likelihood ratio tests to identify branches under positive selection without *a priori* knowledge about which lineages are of interest. Branches identified using this approach were cross-validated using BUSTED (branch-site unrestricted statistical test for episodic diversification) (Pond et al. 2005; Murrell et al. 2015) and using the branch-site likelihood ratio tests from PAML suite (Zhang et al. 2005) (Supplementary Table S6). BUSTED is designed to detect the action of episodic positive selection that is acting on a subset of branches in the phylogeny in at least one site within the alignment (Murrell et al. 2015). The PAML branch-site test compares a model (MA) that allows positive selection on one or more lineages (foreground lineages) with a model (MA1) that does not allow such positive selection. Twice the difference of likelihood for the two models (ΔlnL) is then compared with a χ^2^ distribution with one degree of freedom (Zhang et al. 2005).

To test whether the selection found in the flavivirus phylogenies derived from positive selection or from a relaxation of constraints, the RELAX methodology was applied (Wertheim et al. 2015) (Supplementary Table S6). RELAX calculates a selection intensity parameter, k, by taking into account that, both for sites subjected to purifying selection (ω < 1) and for sites subjected to positive selection (ω > 1), relaxation tends to move ω towards 1. RELAX tests whether selection is relaxed or intensified on a subset of testing branches compared with a subset of reference branches in a predefined tree. In the null model, selection intensity is constrained to 1 for all branches, whereas in the alternative model k is allowed to differ between reference and test groups. The selection on tested branches is intensified or relaxed compared with background branches when *k* > 1 or *k* < 1, respectively.

SLAC, aBSREL, BUSTED, and RELAX analyses were performed either through the DataMonkey server (http://www.datamonkey.org) (Delport et al. 2010) or run locally (through HyPhy, version 2.5.0) (Pond et al. 2005).

To identified sites evolving under positive selection on specific branches, the BEB analysis from MA (with a cutoff of 0.95) was used (Supplementary Table S6).

## 3. Results

### 3.1 MBFVs evolve at similar rates as cISFVs and other members of the *Flaviviridae* family

**Figure 2.**
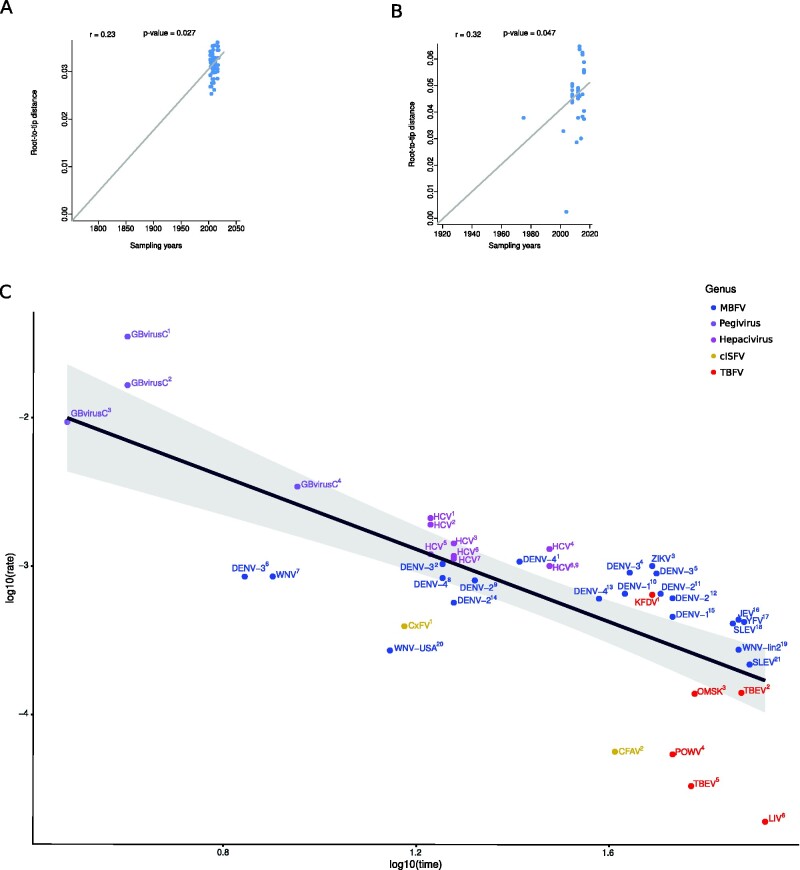
Temporal signal and flavivirus evolutionary rates. Root-to-tip distances as a function of sampling dates are plotted for Asian CxFV (A) and CFAV (B). Each blue dot corresponds to a nucleotide sequence for E; the line is the linear regression calculated using a method that minimizes the residual mean squares. The r coefficient and the corresponding *p* value are also reported. (C) Substitution rate estimates for members of the *Flaviviridae* family. log10-transformed rates are plotted against the log10-transformed time-frames over which they are measured (Supplementary Table S4). A linear regression line (black) with confidence intervals (gray shadow) is shown. Viral genera are colored as per legend. Superscript numbers refer to Supplementary Table S4.

It was previously reported that viruses that infect both vertebrate and invertebrate hosts evolve slower (i.e., have lower substitution rates) than viruses that replicate in a single host or in closely related species (Jenkins et al. 2002). In the context of flavivirus evolution, it is thus expected that MBFVs and TBFVs display lower substitution rates than cISFVs, which only replicate in insect cells. Whereas several studies have investigated MBFVs and TBFVs, limited information is available on the substitution rates of cISFVs. We thus retrieved coding sequence data for the envelope (E), ns3, and ns5 proteins of CFAV, as well as for the E protein of CxFV (Supplementary Table S1). Only sequences with known collection date were included. Indeed, these two viruses were selected because a sufficient number of sequences were available with sampling dates spanning a few decades. For CxFV E coding sequences, we separately analyzed the two major clades (Asia/USA genotype and Africa/Caribbean/Latin America genotype) (Bittar et al. 2016; Miranda et al. 2019). GARD identified no recombination breakpoints in the alignments and maximum likelihood phylogenetic trees were constructed. We next checked for the presence of a temporal signal by performing regression of root-to-tip genetic distances against sampling dates. The date randomization test was used to assess statistical significance (Murray et al. 2016). These analyses revealed a significant temporal signal for the Asian/USA CxFV genotype (Fig. 2A), but not for the African genotype. This is most likely because fewer sequences (*n* = 47) with a shorter time-span among sampling dates (nine years) were available for the African genotype compared to the Asian/USA genotype (seventy-seven sequences, sampled over fifteen years). A significant temporal signal was also detected for the CFAV E phylogeny, although one clear outlier was observed and removed for further analyses (Fig. 2B). Conversely, despite a similar sampling timeframe as for E (approximately forty years), no temporal signal was evident for the CFAV ns5 and ns3 phylogenies (data not shown); this is most likely due to the limited genetic diversity of these gene regions. We thus used a Bayesian approach to estimate substitution rates for the E phylogenies of CxFV (Asian/USA genotype) and CFAV. Rates resulted equal to 3.94 × 10^−4^ (confidence interval (CI): 2.11 × 10^−4^–5.91 × 10^−4^) substitutions/site/year (s/s/y) (CxFV) and 5.63 × 10^−5^ s/s/y (CI: 1.48 × 10^−6^–1.25 × 10^−4^) (CFAV).

Evidence is growing that the estimated rates of viral evolution scale negatively with the time-frame over which they are measured (Aiewsakun and Katzourakis 2016; Simmonds et al. 2019). Because of this time-dependent rate phenomenon, substitution rates are best compared by taking into account the time span of sequence sampling. We thus retrieved rate estimates for members of the *Flaviviridae* family from previous works (Subbotina and Loktev 2012; McMullen et al. 2013; Karan et al. 2014; Di Giallonardo et al. 2015; Faria et al. 2016; Simmonds et al. 2019; Clark et al. 2020) (Supplementary Table S4) and we plotted log10-transformed rates as a function of log10-transformed timescales (Fig. 2C). As generally observed for viral sequences, the values fit within a line (Aiewsakun and Katzourakis 2016). The substitution rate of CxFV was similar to that of several MBFVs and, even accounting for the time-dependent rate phenomenon, the rate for CFAV was one of the lowest among flaviviruses (Fig. 2C). Whereas members of the *Flaviviridae* family that do not alternate vertebrate and invertebrate hosts (*Hepacivirus* and *Pegivirus* genera) tended to show faster evolution (no data point below the regression line), MBFVs did not display particularly low rates (most of them above the regression line). Also, rates for MBFVs were not lower than for the two cISFVs. Conversely, TBFVs tended to display slower rates (Fig. 2C).

### 3.2 Different levels of constraint shape the long-term evolution of flavivirus groups

Substitution rates as calculated above reflect relatively short-term evolutionary processes. To gain insight into the selective patterns acting over longer time frames, we generated phylogenies for the different flavivirus groups (Fig. 1) and we explored the patterns of coding gene evolution. Flaviviruses encode a single polyprotein, which is cleaved to generate three structural proteins and seven non-structural ones. We thus generated multiple sequence alignments of the complete coding sequences of representative viruses belonging to the MBFV, TBFV, NKVFV, cISFV, and dISFV groups (Supplementary Table S2). Sequences were rigorously filtered to ensure high-quality alignment. Using GARD (Kosakovsky Pond et al. 2006), all alignments were screened for recombination, which was not detected in any case. This is in line with the notion that recombination is uncommon in this viral genus (Twiddy and Holmes 2003). Also, no evidence of substitution saturation was detected by Xia's index (Xia et al. 2003; Xia 2013) (Supplementary Table S3).

**Figure 3.**
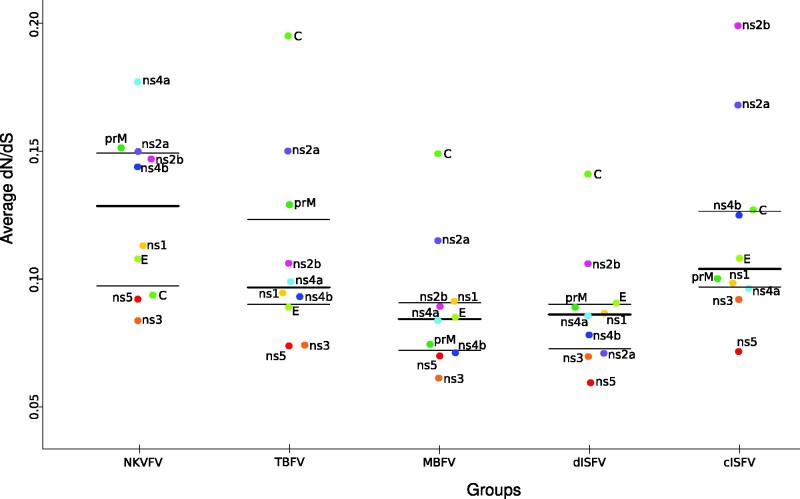
Comparison of dN/dS values. Bee swarm plots comparison of average dN/dS values calculated for all flavivirus proteins.

To obtain an estimate of selective constraint, we used SLAC (Kosakovsky Pond and Frost 2005) to calculate the dN/dS parameter (ratio of the rate of non-synonymous and synonymous substitutions) for all viral proteins (the 2k peptide, which is only 23-bp long, was merged with the ns4a protein) (Supplementary Table S5). Comparisons among flaviviruses indicated that, for most proteins, MBFVs and dISFVs have the lowest dN/dS, whereas NKVFVs display the highest values (Fig. 3). Relatively low values were also observed for TBFVs, with the exclusion of the capsid protein (C), which tended to display fast evolution in this group, as well as in MBFVs and dISFVs (Fig. 3). In all groups, ns5 and ns3 displayed the lowest values, possibly in line with the constraint imposed by the enzymatic activity of the encoded proteins. Interestingly, dISFVs displayed dN/dS values very similar to those of MBFVs, suggesting either that these viruses have recently lost the ability to infect vertebrates (and the relaxation of constraint is still not detectable), or that their vertebrate hosts have not been yet identified. Also, TBFVs showed, on average, less constraint than MBFVs, and dN/dS values were comparable to those of cISFVs. Overall, these results are inconsistent with the idea that host cycling independently drives increased purifying selection (Weaver et al. 1999; Jenkins et al. 2002; Jenkins et al. 2002; Holmes 2003; Greene et al. 2005; Coffey et al. 2008; Vasilakis et al. 2009; Coffey et al. 2013).

### 3.3 Episodic positive selection is common in MBFVs and, to a lesser extent, in TBFVs

We next investigated whether episodic positive selection contributed to the evolution of the five flavivirus groups. As previously mentioned, positive selection can target a minority of residues within a protein that is otherwise constrained. When selection occurs on one or a few branches of a phylogeny, it is said to be episodic and it may underlie the adaptation and emergence of specific viral lineages. To search for evidence of positive selection without an *a priori* hypothesis of which branches may be targeted, we applied aBSREL (adaptive Branch-Site Random Effects Likelihood). Because aBSREL infers probabilistically the number of omega classes for each branch, it is well-suited to analyze branches of different lengths, which may display very different evolutionary patterns. We only analyzed the internal branches of the phylogenies because, compared with external branches, they are expected to be less affected by sequencing errors and to contain fewer transient substitutions.

**Figure 4.**
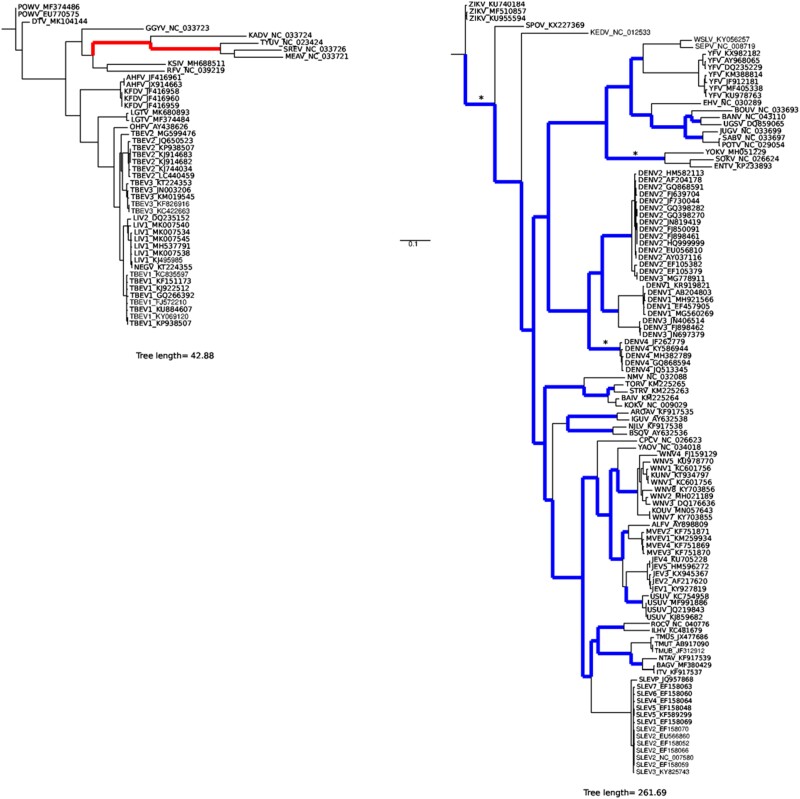
Episodic positive selection in flaviviruses. Phylogenetic trees for (A) TBFV and (B) MBFV. Colored and thick branches indicate evidence of positive selection detected using aBSREL and confirmed by two other methods (BUSTED and the PAML branch-site models). Asterisks denote branches showing evidence of relaxed constraint (as detected by RELAX).

aBSREL detected no evidence of positive selection in the cISFV, and dISFV phylogenies (Supplementary Fig. S2). Conversely, two, three, and forty-two branches in the NKVFV, TBFV, and MBFV trees, respectively, showed evidence of positive selection (Supplementary Table S6). To validate these results, all significant branches were individually tested for evidence of positive selection using the MA/MA1 models implemented in PAML, as well as with BUSTED (Zhang et al. 2005; Murrell et al. 2015). Both methods apply likelihood ratio tests to assess whether one (or more) pre-specified foreground lineage is under positive selection. The two methods did not validate positive selection on the two branches in the NKVFV phylogeny. Conversely, both MA/MA1 and BUSTED confirmed positive selection for two out of three branches in the TBFV tree and thirty-eight out of forty-two branches in the MBFV phylogeny (Supplementary Table S6, Fig. 4 and Supplementary Fig. S2). In the case of TBFVs, both positively selected branches separate viruses that infect seabirds (the so-called seabird clade) (Grard et al. 2007) from all other viruses (Fig. 4A). Conversely, selection tends to be more homogeneously distributed in the MBFV phylogeny (Fig. 4B).

Relaxation of functional constraint along specific branches may sometimes generate spurious evidence of positive selection (Wertheim et al. 2015). To check whether this was the case for the branches, we identified as positively selected in the TBFV and MBFV phylogenies, we applied the RELAX method, which evaluates if selection on one (or more) tested branch (each branch showing evidence of positive selection) is relaxed (i.e., the *k* parameter is <1) compared with background branches (Wertheim et al. 2015). Most tested branches, both in the MBFV and in the TBFV phylogenies, had *k* values >1, and significant evidence of relaxation was obtained for three MBFV branches only (Supplementary Table S6, Fig. 4B). Notably, one of these branches is the one leading to Entebbe bat virus, Yokose virus, and Sokoluk virus, which have no known vector. Overall, these data indicate that although they are generally more constrained than flaviviruses with a single host, MBFVs, and to a lesser extent TBFVs, evolve by frequent episodes of positive selection.

**Figure 5.**
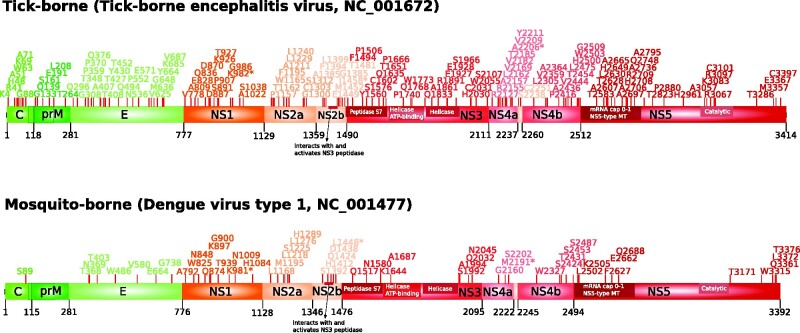
Localization of positively selected sites. Positively selected sites (red sticks) are mapped onto a schematic representation of TBFV and MBFV polyproteins.

We next aimed to determine the overall fraction of positively selected sites and to investigate whether specific proteins or regions are common targets of positive selection. We thus identified positively selected sites using the BEB analysis from the MA model and by imposing a 0.95 significance cutoff. We detected 136 (4.18%) and 59 (1.82%) positively selected sites in the TBFV and MBFV phylogenies, respectively (Supplementary Table S6, Fig. 5). This clearly implies that even if few lineages evolve by positive selection in TBVFs, the selective pressure is very strong.

Both in TBFVs and in MBFVs, positively selected sites tended to be scattered along the polyprotein region and all proteins, with the exclusion of prM (membrane) in MBFVs, showed at least one positively selected site (Fig. 5). However, TBFVs tended to display more selected sites in the structural proteins (4.98%) compared with the non-structural ones (3.96%), whereas the opposite was true for MBFVs (structural, 1.13%; non-structural, 2.02%). Thus, episodic positive selection drove the evolution of most protein products in MBFVs and TBFVs.

## 4. Discussion

Flaviviruses are an extremely diversified viral family, with different transmission routes and host ranges. Despite sharing an overall similar genetic structure, these viruses have diverse epidemiological and ecological features. MBFVs and TBFVs have been widely studied, as they represent prevalent pathogens for humans and domestic animals (Pierson and Diamond 2020). Insect-specific flaviviruses have also gained attention in recent years, due to the ability of some ISFVs to enhance or suppress the replication of dual-host flaviviruses in co-infected mosquitoes (Blitvich and Firth 2015; Baidaliuk et al. 2019). Notably, these viruses are regarded as a possible strategy to control mosquito populations and consequently limit disease transmission (Blitvich and Firth 2015).

Viruses that alternate among taxonomically distant hosts are generally considered to be slow-evolving and highly constrained by the need to respond to distinct selective environments (Weaver et al. 1999; Jenkins et al. 2002; Jenkins et al. 2002; Holmes 2003; Greene et al. 2005; Coffey et al. 2008, 2013; Vasilakis et al. 2009). Nevertheless, these viruses show a remarkable propensity to emerge in new geographic areas and to adapt to novel hosts and/or vectors (Farajollahi et al. 2011; Marr and Cathey 2013; Fredericks and Fernandez-Sesma 2014; Abushouk et al. 2016; Pierson and Diamond 2020). Indeed, host switches have been common during the evolution of flaviviruses (Geoghegan et al. 2017). Conversely, less is known about the evolution of the other flavivirus groups, and the availability of sequence data is much more limited compared with MBFVs and TBFVs. However, enough sequence information is present in databases for CFAV and CxFV to allow the construction of phylogenetic trees that show a temporal signal, thus allowing the application of molecular clock models to estimate evolutionary rates. These viruses offer a good comparison to dual-host flaviviruses, as they are genetically related but do not alternate between different hosts. Comparison of evolutionary rates was performed by taking into account the time-frame of measurement. This is expected to result in more reliable comparisons, as the time-dependency of substitution rates is a general phenomenon for viral (and non-viral) species (Aiewsakun and Katzourakis 2016; Simmonds et al. 2019). It follows that rates *per se* are not very informative of the evolutionary process. Our data indicate that CFAV and CxFV display relatively different substitution rates. This might depend on several factors, including features specific of their main hosts (*Aedes* spp. for CFAV and *Culex* spp. for CxFV) (Grubaugh et al. 2016a) or transmission modes (horizontal or vertical), these latter still poorly investigated (Blitvich and Firth 2015; Agboli et al. 2019). Nevertheless, these data, together with estimates obtained for single-host members of the *Flaviviridae* family, indicate that MBFVs do not display unusually slow evolution. Conversely, TBFVs, with the exclusion of Kyasanur forest disease virus, displayed some of lowest substitution rates among flaviviruses. These data are in agreement with the view that the genetic diversification of TBFVs mainly occurs during horizontal transmission to the vertebrate hosts (Grubaugh et al. 2016b). However, due to the life cycle of ticks, which takes approximately three years to complete, such transmissions occur infrequently, eventually resulting in slow viral evolution (Grubaugh et al. 2016b; Vechtova et al. 2020). Moreover, transstadial transmission in ticks most likely imposes additional bottlenecks, although available evidence suggests that these are relaxed (Grubaugh et al. 2016b). On the one hand, these results suggest that flavivirus evolutionary rates are not mainly determined by the alternation between vertebrate and invertebrate hosts, but rather by the specific nature and ecological characteristics of individual hosts, as well as by the number of transmissions. On the other hand, we found that flaviviruses that only infect vertebrates (i.e. hepaciviruses and pegiviruses) tend to evolve faster than cISFVs, MBFVs, and TBFVs. Thus, whereas the cycling between taxonomically different hosts may not explain low evolutionary rates, these might be associated with the infection of invertebrates. Also, we cannot exclude that host alternation does add some level of constraint and that different constraints play out for cISFVs. For instance, vertical transmission, which is thought to represent a major mechanism by which cISFVs persist in mosquitoes in nature (Blitvich and Firth 2015), might impose tighter bottlenecks than horizontal transmission (see also below).

We should add that our analysis of evolutionary rates has some caveats. First, data for cISFVs refer to two viruses only and clearly represent estimates of the actual rates. Second, for both viruses, we only analyzed the E protein, as a sizable number of CxFV sequences were available only for this protein, whereas the ns3 and ns5 datasets of CFAV did not hold sufficient temporal signal. Indeed, even for the CFAV E protein the temporal signal was not particularly strong, with a borderline *p* value. This is most likely due to the fact that the number of available sequences is low (less than fifty) and the timeframe relatively narrow (approximately forty years). Whatever the underlying reasons, we limited our analysis to the E protein of both viruses, although we found clear differences in the evolutionary patterns among viral proteins (Fig. 3). Nonetheless, it is worth mentioning that for all flavivirus groups, dN/dS estimates for E were very close to the median (Fig. 3) and that the majority of previous rate estimates, which we used to obtain the regression line, were derived from the E protein (Supplementary Table S4). Finally, some differences across previous studies are evident from our analysis (Fig. 2C). Such discrepancies are likely due to many factors, including the sequence sampling scheme, the genomic region used for the analysis, and the methodology applied to infer substitution rates. Moreover, some estimates might rely on poorly informative phylogenies. For instance, Clark and coworkers indicated that the temporal signal for their TBEV dataset was weak and the one for louping ill virus was even weaker, making rate estimates not fully reliable (Clark et al. 2020). However, although some estimates may suffer from similar or other problems, they showed an overall good fit to the regression line. Thus, although a larger number of sequence data, for CxFV, CFAV, and for other ISFVs, will be required to gain a full picture of insect-specific flavivirus evolution, we consider that our data allow an accurate, albeit preliminary comparison of evolutionary rates among flaviviruses.

The evolutionary rates we analyzed above refer to relatively short-term processes and they are limited to the analysis of single viral species over a few decades. Moreover, such analyses cannot be performed for viruses with limited sampling. To gain a better understanding of the long-term selective processes acting on flaviviruses—i.e. over the time-frame of viral species emergence (since ∼15,000 years ago) (Moureau et al. 2015)—we analyzed viral phylogenies representative of the five flavivirus groups. All these are monophyletic, with the exclusion of dISFVs (Blitvich and Firth 2015). Results indicated that at the coding sequence level, MBFVs and dISFVs evolve under the strongest purifying selection. However, while TBFVs and cISFVs do not show extremely different levels of constraint, the former alternate among hosts whereas the latter do not. NKVFVs clearly displayed the lowest level of purifying selection. We nonetheless note that data on these viruses derive from a limited number of sequences (*n* = 7) and might thus suffer from biases related to sample size and sequencing errors. Again though, these data suggest that the level of purifying selection is mainly dictated by factors other than alternation among hosts.

At the level of single proteins, there were commonalities and differences among flavivirus groups. As mentioned above, ns3 and ns5 showed the lowest dN/dS in all groups, reflecting strong selective constraint. Conversely, the capsid protein had high dN/dS in MBFVs, TBFVs, and dISFVs, but not in the other groups. The main function of capsid is the packaging of the viral genome, but the protein is multifunctional and interacts with a number of host factors (Sotcheff and Routh 2020), possibly suggesting that its relatively faster evolution in dual-host flaviviruses is driven by the need to adapt to distinct cellular environments. However, our positive selection analysis did not reveal major signatures for capsid, suggesting that high dN/dS is more the result of relaxed constraint than of adaptive evolution.

Several studies have analyzed the degree of selective pressure exerted by different hosts on flavivirus evolution. Albeit controversial (Nelson et al. 2018; Bialosuknia et al. 2019), the strength of intra-host purifying selection acting on WNV was reported to be stronger in birds than in mosquitoes and to even differ among mosquito and bird species (Jerzak et al. 2008; Grubaugh et al. 2015; Grubaugh and Ebel 2016). Specifically, WNV passaging in highly susceptible hosts (e.g. crows) was associated with mutational tolerance and weaker purifying selection (Grubaugh et al. 2015). Conversely, transmission experiments with Powassan virus (POWV) indicated that purifying selection is more efficient in the vector (*Ixodes scapularis)* than in the mammalian host (mouse) (Grubaugh et al. 2016b). It is thus possible that specific, ecologically relevant hosts, either vertebrate or invertebrate, play a major role in determining the overall level of purifying selection acting on MBFVs and TBFVs. Clearly, in the case of cISFVs, the selective pressure can only be exerted by the arthropod host and most of these viruses have been isolated from mosquitoes, which seem to exert relatively modest pressure. However, measures of selective pressure were obtained for WNV and passaging of the virus in different mosquito species indicated that the overall intra-host selective pattern is dictated by virus-vector interactions (Grubaugh et al. 2015). Moreover, MBFV transmission to vertebrates requires infection of the mosquito midgut and salivary glands, both of which subject the virus to severe bottlenecks (Grubaugh et al. 2016a). This might explain why experimental infection in birds (i.e. bypassing the vector stage) results in lower genetic diversity than in vectors (Deardorff et al. 2011; Grubaugh et al. 2015), whereas similar or even lower levels of selective constraint were observed in naturally infected birds compared with mosquitoes (Nelson et al. 2018; Bialosuknia et al. 2019; Caldwell et al. 2020). cISFVs are not expected to face the same anatomical barriers as MBFVs, but most likely experience bottlenecks during vertical (e.g. transovarial) transmission (Blitvich and Firth 2015). Further studies will thus be required to clarify the selective events acting on insect-specific flaviviruses.

Of course, purifying selection is not the only aspect of the evolutionary process. In the above-mentioned experiments with WNV in birds, Grubaugh and coworkers found that the strength of both purifying and positive selection within hosts were stronger in the less susceptible bird species (robins) (Grubaugh et al. 2015). This clearly exemplifies the concept that positive selection can act on a minority of sites against a background of purifying selection. Indeed, we found ample evidence that episodic positive selection drove the evolution of MBFVs, despite their high constraint. Positive selection was also detected on two branches of the TBFVs phylogeny, which define the seabird clade. Thus, positive selection was much more common during the evolution of arthropod-borne flaviviruses than previously thought. Conversely, we did not detect any evidence of positive selection in the other flavivirus groups.

The approach we applied to detect positively selected branches is highly robust and it is based on the intersection of three methods, as well as on a further check that relaxation of constraint is not misinterpreted as positive selection. Interestingly, we found that one of the branches of the MBFVs phylogeny that experienced a relaxation of purifying selection was the one leading to the three viruses that have no known arthropod host. The reason for this observation might relate to the release of these viruses from the alternation of infections in vertebrates and invertebrates (although data above do not strongly support this view), from the absence of an arthropod host (although mosquitoes do not seem to exert a strong selective pressure), or from the fact that mammals exert weak selective pressures, as previously shown for Powassan virus (a TBFVs) and suggested by the higher diversity of DENV in humans compared with *A. aegypti* (Lin et al. 2004; Sim et al. 2015).

It is presently unclear why the branches separating the seabird viruses from the other TBFVs show such strong evidence of positive selection, with a large number of positively selected sites. The explanation may lie in the adaptation to a new arthropod vector (soft vs hard ticks), to a new vertebrate host (mainly mammals vs birds) or both. Conversely, the MBFV phylogeny is characterized by evidence of positive selection on several branches without any apparent preference for specific lineages or clades. Instances of positive selection at specific sites associated with higher transmission were previously reported for MBFVs and other arthropod-borne viruses (Moudy et al. 2007; Brault et al. 2007; Tsetsarkin et al. 2007; May et al. 2011; McMullen et al. 2011; Tsetsarkin et al. 2014; Stapleford et al. 2014). Positive selection does not necessarily favor infection in one host with a loss of fitness in another. For instance, in the case of ZIKV, a mutation in the ns1 protein that occurred in the Asian lineage increases infectivity in mosquitoes and also allows evasion of mammalian innate immune responses (Liu et al. 2017; Xia et al. 2018). We found that ns1 evolved under positive selection in TBFVs and MBFVs, but, overall, we detected no preferential selection target among flavivirus proteins, with positively selected sites scattered along the polyprotein sequence. This observation also speaks against the possibility that the signatures of positive selection are secondary to mutations that arise to evade RNAi. In fact, at least in WNV and POWV, specific regions are preferentially targeted by virus-derived small RNAs (Brackney et al. 2015; Grubaugh et al. 2016b). Moreover, RNAi does not preferentially generate non-synonymous diversity. Rather, it promotes diversity at both synonymous and non-synonymous sites, the fate of which is then dictated by natural selection.

In summary, we provide evidence that the trajectories of flavivirus evolution are complex and most likely determined by multiple factors, not limited to the alternation between taxonomically divergent hosts. The frequency of both positive and purifying selection, especially in MBFVs, suggests that a minority of sites in the viral polyprotein experience weak constraint and can evolve to generate new viral phenotypes and possibly promote adaptation to new hosts. This might help explain the common observation that flaviviruses can rapidly emerge and spread in multiple locations and invade new ecological niches. We note, however, that a limitation of our study is that evolutionary analyses were based on consensus sequences, whereas flaviviruses exist within hosts as viral swarms. Intra-host diversity is still poorly analyzed, but recent data on WNV (Caldwell et al. 2020) indicated that consensus sequence evolution does not directly correlate with intra-host variability. Thus, phenotypically relevant minority variants may exist in the viral swarm and never be reflected in consensus sequences (Caldwell et al. 2020). This is relevant because the composition and breadth of the intra-host viral population can modulate viral phenotypes, including fitness, virulence, and immune escape, eventually facilitating adaptation to diverse hosts and ecosystems (Domingo et al. 2012; Dolan et al. 2018; Lauring 2020). A deeper understanding of flavivirus evolution will thus require the joint analysis of inter- and intra-host viral diversity. This is especially true for ISFVs, for which no data of intra-host diversity is presently available.

## Funding

This work was supported by the Italian Ministry of Health, “Ricerca Corrente 2019–2020” to M.S., “Ricerca Corrente 2018–2020” to D.F., and “Ricerca Corrente 2021” to M.S.

## Data accessibility

Lists of Flavivirus accession IDs are reported in Supplementary Table S1 and Supplementary Table S2. All of the sequences were retrieved from the NCBI (http://www.ncbi.nlm.nih.gov/) and ViPR (Virus Pathogen Resource, https://www.viprbrc.org/) databases.

## Supplementary data

Supplementary data are available at *Virus Evolution* online.

**Conflict of interest**: None declared.

## Supplementary Material

veab040_Supplementary_Data
