# Supplementary material for: Alternation between taxonomically divergent hosts is not the major determinant of flavivirus evolution
Source: Virus Evol. 2021 Apr 21;7(1):veab040. doi: 10.1093/ve/veab040 (PMC8093920; doi:10.1093/ve/veab040)
Supplement: veab040_Supplementary_Data [file veab040_supplementary_data.pdf]

Alternation between taxonomically divergent hosts is not the major determinant of flavivirus evolution.

Chiara Pontremoli[1]*, Diego Forni[1], Mario Clerici[2,3], Rachele Cagliani[1], Manuela Sironi[1]

[1] Scientific Institute IRCCS E. MEDEA, Bioinformatics, 23842 Bosisio Parini, Italy;

[2] Department of Physiopathology and Transplantation, University of Milan, 20122 Milan, Italy;

[3] Don C. Gnocchi Foundation ONLUS, IRCCS, 20121 Milan, Italy.

*** Corresponding Author:** Bioinformatics - Scientific Institute IRCCS E. MEDEA, Via Don L. Monza 20, 23842 Bosisio Parini, Italy; telephone: +39031877826; e-mail: chiara.pontremoli@lanostrafamiglia.it

Supplementary Table S1. List of cell fusing agent (CFAV) and Culex flavivirus (CxFV) sequences used for dating analysis.

CxFV -Asia/USA genotype- *E* gene (n=77)		CxFV -Africa/Caribbean/Latin America genotype- *E* gene (n=47)		CFAV (n=48) *ns3* gene		CFAV (n=48) *ns5* gene		CFAV (n=49) *E* gene	
**IDs**	**Dates**	**IDs**	**Dates**	**IDs**	**Dates**	**IDs**	**Dates**	**IDs**	**Dates**
Q518484	2006	JX897904	2010	AB813755	2012	AB813784	2012	AB488425*	2004
JF938690	2006	JX416692	2010	AB813756	2012	AB813785	2012	AB813726	2012
KM655810	2009	JX416696	2011	AB813757	2012	AB813786	2012	AB813727	2012
JQ065880	2011	JX416697	2012	AB813758	2012	AB813787	2012	AB813728	2012
JQ065879	2011	JX416695	2010	AB813759	2012	AB813788	2012	AB813729	2012
JQ308187[+]	2010	JX416694	2010	AB813760	2012	AB813789	2012	AB813730	2012
KM655811	2012	JX416693	2010	AB813761	2012	AB813790	2012	AB813731	2012
KM655813	2012	GU289697	2008	AB813762	2008	AB813791	2008	AB813732	2012
KM655812	2004	GU289688	2008	AB813763	2012	AB813792	2012	AB813733	2008
JQ308186[+]	2010	GU289686	2008	AB813764	2012	AB813793	2012	AB813734	2012
HQ678513[+]	2009	MH719098[+]	2008	AB813765	2012	AB813794	2012	AB813735	2012
FJ663030	2007	EU879060[+]	2007	AB813766	2012	AB813795	2012	AB813736	2012
FJ663028	2007	GU289683	2008	AB813767	2012	AB813796	2012	AB813737	2012
HQ634589	2009	GU289687	2008	AB813768	2008	AB813797	2008	AB813738	2012
FJ502998	2008	GU289700	2008	AB813769	2012	AB813798	2012	AB813739	2008
FJ502996	2008	GU289692	2008	AB813770	2012	AB813799	2012	AB813740	2012
FJ503001	2008	GU289685	2008	AB813771	2012	AB813800	2012	AB813741	2012
FJ502997	2008	GU289699	2008	AB813772	2012	AB813801	2012	AB813742	2012
FJ503000	2008	GU289690	2008	AB813773	2008	AB813802	2008	AB813743	2012
HQ634593	2009	GU289695	2008	AB813774	2008	AB813803	2008	AB813744	2008
KX512322	2016	GU289694	2008	AB813775	2008	AB813804	2008	AB813745	2008
FJ663031	2007	GU289691	2008	AB813776	2008	AB813805	2008	AB813746	2008
FJ502999	2008	GU289693	2008	AB813777	2008	AB813806	2008	AB813747	2008
HQ634590	2009	GU289684	2008	AB813778	2008	AB813807	2008	AB813748	2008
FJ502995[+]	2008	GU289696	2008	AB813779	2008	AB813808	2008	AB813749	2008
FJ663026	2007	GU289698	2008	AB813780	2008	AB813809	2008	AB813750	2008
FJ663032	2007	GU289689	2008	AB813781	2008	AB813810	2008	AB813751	2008
FJ663029	2007	KM081646	2012	AB813782	2008	AB813811	2008	AB813752	2008
FJ663033	2007	KM081642	2012	AB813783	2008	AB813812	2008	AB813753	2008
FJ663027	2007	KM081643	2012	GQ165810[+]	2002	GQ165810[+]	2002	AB813754	2008
MH188006[+]	2016	KM081644	2012	KJ476731[+]	2011	KJ476731[+]	2011	GQ165810[+]	2002
MK609523	2018	KM081645	2012	KJ741267[+]	2012	KJ741267[+]	2012	KJ476731[+]	2011

MK609522	2018	KM081641	2012	LC496857[+]	2016	LC496857[+]	2016	KJ741267[+]	2012
MK609521	2018	EU805805	2006	LR596014[+]	2013	LR596014[+]	2013	LC496857[+]	2016
MG673528	2016	KC700042	2009	LR694072[+]	2016	LR694072[+]	2016	LR596014[+]	2013
MG673529	2016	KC700041	2009	LR694073[+]	2016	LR694073[+]	2016	LR694072[+]	2016
MG673526	2016	KC700043	2009	LR694074[+]	2015	LR694074[+]	2015	LR694073[+]	2016
MN318426[+]	2017	MT197496[+]	2017	LR694075[+]	2014	LR694075[+]	2014	LR694074[+]	2015
MG673527	2016	KY349933[+]	2013	LR694076[+]	2016	LR694076[+]	2016	LR694075[+]	2014
MG673530	2016	KT726939[+]	2012	LR694077[+]	2015	LR694077[+]	2015	LR694076[+]	2016
JQ308188[+]	2010	FJ503003	2008	LR694078[+]	2015	LR694078[+]	2015	LR694077[+]	2015
LC513839[+]	2017	FJ503002	2008	LR694079[+]	2013	LR694079[+]	2013	LR694078[+]	2015
AB701776[+]	2008	KU726615[+]	2009	LR694080[+]	2013	LR694080[+]	2013	LR694079[+]	2013
NC_008604[+]	2003	KC700045	2009	LR694081[+]	2016	LR694081[+]	2016	LR694080[+]	2013
AB377213[+]	2003	LC504568[+]	2016	MH237596[+]	2016	MH237596[+]	2016	LR694081[+]	2016
KX657782	2016	MN294938[+]	2016	MH310082[+]	2015	MH310082[+]	2015	MH237596[+]	2016
AB262760	2003	GQ165808	2008	MK860761[+]	2013	MK860761[+]	2013	MH310082[+]	2015
AB262765	2003			NC_001564[+]	1975	NC_001564[+]	1975	MK860761[+]	2013
AB701769[+]	2007							NC_001564[+]	1975
AB262761	2003								
AB701775[+]	2006								
AB701770[+]	2004								
AB701774[+]	2005								
HQ634592	2009								
FJ663034[+]	2007								
HQ634591	2009								
AB262762	2003								
KX924633[+]	2016								
AB701772[+]	2005								
AB701773[+]	2005								
AB701768[+]	2006								
AB701767[+]	2005								
AB701771[+]	2004								
AB701766[+]	2004								
AB262767	2004								
AB262763	2003								
AB262764	2004								
AB262766	2004								
JQ065883	2011								
JQ308190[+]	2010								

KM655815	2012								
KM655814	2012								
JQ308189+	2010								
JQ065878	2011								
JQ065882	2011								
JQ065877	2011								
JQ065881	2011								

*outlier; +complete genome

Supplementary Table S2. List of flavivirus strains used in the positive selection analyses.

Phylogenetic group/ Virus specie	Strain Name	GenBank Accession	Collection Date	GenBank Host	Country
**Tick-borne flaviviruses -TBFV- (n=46)**					
**Kyasanur Forest disease virus**	P9605	JF416958=HM055369	1957	*Homo sapiens*	India
	G11338	JF416959	1957	*Haemaphysalis spinigera*	India
	W-377	JF416960	1957	*Semnopithecus entellus*	India
**Alkhumra hemorrhagic fever virus**	Zaki #2	JX914663	2010	*Homo sapiens*	Egypt
	3B1	JF416961	2009	*Hyalomma dromedarii*	Saudi Arabia
**Omsk hemorrhagic fever virus**	Kubrin	AY438626	1947	*Homo sapiens*	Russia
**Powassan virus**	Spassk-9	EU770575	04/15/1975	*Dermacentor silvarum*	Russia
	L. B.	MF374486	09/23/1958	*Homo sapiens*	Canada
**Deer tick virus**	strain MeC17-166	MK104144	2017	*Ixodes scapularis*	USA
**Royal Farm virus**	Afghanistan	NC_039219=DQ235149	1972	*Argas hermanni*	Afghanistan
**Karshi virus**	LEIV-7192 Tur	MH688511	Ap 2016	*Hyalomma asiaticum*	China
**Tick-borne encephalitis virus**	Kubinova	KJ922512	1953	*Homo sapiens*	Czech Republic
	AS33	GQ266392	2005	*Ixodes ricinus*	Germany
	Salem	FJ572210	2006	*Macaca sylvanus*	Germany
	Leila-BH95/15	KU884607	05/2015	*Ovis aries*	Germany
	118-71	KY069120	1971	*Spermophilus undulatus*	Russia
	A104	KF151173	1990	*Apodemus flavicollis*	Austria
	CGl223	KC835597	1990	*Myodes glareolus*	Slovakia
	Sorex 18-10	KP938507	2010	*Sorex sp.*	Russia
	Senzhang	JQ650523	1953	*Homo sapiens*	China
	Himalaya-1	MG599476	07/2013	*Marmota himalayana*	China
	Sapporo-17-Io1	LC440459	2017	*Ixodes ovatus*	Japan
	Tomsk-M83	KJ739731	2006	*Sorex araneus*	Russia
	Malishevo	KJ744034	1978	*Aedes vexans nipponii*	Russia
	Tomsk-M202	KJ914683	07/10/2008	*Microtus arvalis*	Russia
	Tomsk-PT14	KJ914682	04/15/2008	*Pica pica*	Russia
	Aina	JN003206	1963	*Homo sapiens*	Russia
	LEIV-10133Al	KT224353	1984	*Ixodes persulcatus*	Russia
	Tomsk-PT122	KM019545	07/15/2006	*Acrocephalus dumetorum*	Russia
	Zabaikalye 68B-00	KC422663	2000	*Clethrionomys rutilus*	Russia
	Sakhalin_6-11	KF826916	2011	*mosquitoes pool*	Russia
**Negishi virus**	Negishi	KT224355	1948	*Homo sapiens*	Russia
**Langat virus**	TP64	MF374484	04/18/1956	*Homo sapiens*	Malaysia
	T 1674	MK680893	Sett 1973	*Haemaphysalis*	Thailand

				papuana	
**Louping ill virus**	LIV/Dog	MH537791	2015	*Canis lupus familiaris*	United Kingdom
	Primorye-185-91	KJ495985	07/22/1991	*Homo sapiens*	Russia
	SCO_G_1979	MK007545	1979	*Sus scrofa domesticus*	United Kingdom: Scotland
	SCO_K_1980	MK007538	1980	*Lagopus lagopus scotica*	United Kingdom: Scotland
	IRE_IRE2_1971	MK007540	1968	*Ixodes ricinus*	United Kingdom: Ireland
	ENG_PEN4_1983	MK007534	1983	*Ovis aries*	United Kingdom
	87/2617	DQ235152	1987	*Ovis aries*	Spain
**Gadgets Gully virus**	CSIRO122	NC_033723=DQ235145	1976	*Ixodes ricinus*	Australia, Macquarie Island
**Meaban virus**	Brest/Ar/T70	NC_033721=DQ235144	1981	*Ornithodoros maritimus*	France
**Saumarez Reef virus**	CSIRO 4	NC_033726=DQ235150	1975	*Ornithodoros capensis*	Australia
**Tyuleniy virus**	LEIV-6C	NC_023424=KF815939	1969	*Ixodes uriae*	Russia
**Kadam virus**	Amp 6640	NC_033724=DQ235146	1967	*Rhipicephalus pravus*	Uganda
Mosquito-borne flaviviruses -MBFV- (n=110)					
**Aroa virus**	Macaray 01809	KF917535	1975	*Cricetulus aureus*	Venezuela
**Bussuquara virus**	BeAn 4073	AY632536= NC_009026.2	1956	*Alouatta belzebul*	Brazil
**Iguape virus**	SPAn 71686	AY632538	Luglio 1994	*Anopheles cruzii*	Brazil
**Naranjal virus**	25008	KF917538	1976	*Sentinel hamster*	Ecuador
**Dengue virus 1**	TSV08	KR919821	2008	*Homo sapiens*	Australia
	D1/hu/Yap/ NIID27/2004	AB204803	2004	*Homo sapiens*	Japan
	P72-1244	EF457905	1972	*Homo sapiens*	Malaysia
	PNG 2015c	MH921566	2015	*Homo sapiens*	Australia
	P1253/China/GD/ CZ/2014	MG560269	10/16/2014	*Homo sapiens*	China
**Dengue virus 2**	DENV-2/KH/BID-V2019/2001	JF730044	2001	*Homo sapiens*	Cambodia
	DENV-2/PR/2DN/ 1994	GQ398270	1994	*Homo sapiens*	Puerto Rico
	DENV-2/PR/35DN/ 1994	GQ398282	1994	*Homo sapiens*	Puerto Rico
	DENV-2/BR/BID-V2377/2000	JN819419	2000	*Homo sapiens*	Brazil
	DENV-2/BR/BID-V2399/2007	FJ850091	2007	*Homo sapiens*	Brazil
	DENV-2/BZ/BID-V2952/2002	FJ898461	2002	*Homo sapiens*	Belize
	DENV-2/GU/FDA-GUA09/2009	HQ999999	2009	*Homo sapiens*	Guatemala
	DENV-2/TH/BID-	GQ868591	1964	*Homo sapiens*	Thailand

	V3357/1964				
	DENV-2/KH/BID-V2036/2003	FJ639704	2003	*Homo sapiens*	Cambodia
	43	AF204178	1987	*Homo sapiens*	China
	D2/TO/UH20/1974	HM582113	1974	*Homo sapiens*	Tonga
	1349	EU056810	1983	*Homo sapiens*	Burkina Faso
	TSV01	AY037116	1993	*Homo sapiens*	Australia
	Dak Ar 2039	EF105382	1980	*Aedes luteocephalus*	Burkina Faso
	P8-1407	EF105379	1970	*Sentinel monkey*	Malaysia
**Dengue virus 3**	HN201705	MG778911	07/21/2017	*Homo sapiens*	China
	Cairns 98	JN406514	1998	*Homo sapiens*	Australia
	DENV-3/AI/BID-V2976/2001	FJ898462	2001	*Homo sapiens*	Anguilla
	DENV3/BR/D3LIMHO/2006	JN697379	2006	*Homo sapiens*	Brazil
**Dengue virus 4**	H781363	JQ513345	03/18/2011	*Homo sapiens*	Brazil
	PNG 2016a	MH382789	2016	*Homo sapiens*	Australia
	Ser4_Thailand_Bangkok_Seq81	KY586944	2001	*Homo sapiens*	Thailand
	P75-514	JF262779	1975	*sentinel monkey*	Malaysia
	DENV-4/PH/BID-V3361/1956	GQ868594	1956	*Homo sapiens*	Philippines
**Cacipacore virus**	BeAn 3276000	KF917536=NC_026623	1977	*Formicarius analis*	Brazil
**Japanese encephalitis virus**	ME802	KY927819	05/19/2013	*Homo sapiens*	Cambodia
	FU	AF217620	1995	*Homo sapiens*	Australia
	C17	KX945367	03/16/2016	*Homo sapiens*	Angola
	VN 113	KU705228	1979	*Homo sapiens*	Viet Nam
	Muar	HM596272	1952	*Homo sapiens*	Malaysia
**Koutango virus**	PM148	MN057643	2016	*Phlebotominae*	Niger
**Alfuy virus**	MRM3929	AY898809	1966	*Centropus phasianinus*	Australia
**Murray Valley encephalitis virus**	V11-10	KM259934	04/2008	*Homo sapiens*	Australia
	611W/WA/08	KF751871	1973	*Culex annulirostris*	Australia
	OR156	KF751870	1956	*Homo sapiens*	Papua New Guinea
	NG156	KF751869	1966	*mixed Culicines*	Papua New Guinea
**St. Louis encephalitis virus**	72 V 4749	EF158069	1972	*Culex tarsalis*	USA
	Parton	EF158070	1933	*Homo sapiens*	USA
	V 2380-42	EF158052	2001	*Culex quinquefasciatus*	USA
	Kern217	NC_007580=DQ525916=EF158058	1989	*Culex tarsalis*	USA
	65 V 310	EF158059	1961	Bird	Mexico
	Hubbard	EU566860	1937	*Homo sapiens*	USA
	GHA-3	EF158066	1955	*Butorides virescens*	Haiti
	USA/CA/2016/	KY825743	09/09/2016	*Homo sapiens*	USA

	human/UC-1				
	GML 902612	EF158064	1973	*Haemagogus equinus*	Panama
	FLU3632	KF589299	03/27/2006	*Homo sapiens*	Peru
	BeAr 23379	EF158048	1960	*Sabethes belisarioi*	Brazil
	GML 903797	EF158060	1983	*Sentinel chicken*	Panama
	CorAn 9124	EF158063	1966	*Calomys musculinus*	USA
	Palenque-C475	JQ957868	2008	*Culex nigripalpus*	Mexico
**Usutu virus**	Meise H	JQ219843	08/29/2002	*Parus caeruleus*	Austria
	BD1/17-AT	MF991886	07/24/2017	*Homo sapiens*	Austria
	ArB1803	KC754958	1969	*Culex perfuscus*	Central African Republic
	BAT1USUTU-BNI	KJ859682	2013	*Pipistrellus pipistrellus*	Germany
**Kunjin virus**	MRM5373	KT934797	1991	*Homo sapiens*	Australia
**West Nile virus**	ArEq001	KC601756	02/2011	*Homo sapiens*	India
	1048813	GQ851602	1960	*Culex annulirostris*	Australia
	MRM16	MH021189	10/2017	*Homo sapiens*	Belgium
	WNV/Belgium/ 2017/Antwerpen	DQ176636	1978	*Coracopsis vasa*	Madagascar
	Madagascar-AnMg798	FJ159129	2006	*Uranotaenia unguiculata*	Russia
	101_5-06-Uu	KU978770	02/12/1988	*Homo sapiens*	India
	I 804994	KY703855	1993	*Rhipicephalus guilhoni*	Senegal
	ArD96655/1993/SN	KY703856	1992	*Culex perfuscus*	Senegal
**Yaounde virus**	Dak Ar Y276	NC_034018	NA	NA	Cameroon
**Bainyik virus**	MK7979	KM225264	1966	*Aedes* sp.	Papua New Guinea
**Kokobera virus**	AusMRM 32	NC_009029=AY632541	1960	*Culix annulirostris*	Australia
**New Mapoon virus**	CY1014	NC_032088=KC788512	1998	Mosquito	Australia
**Stratford virus**	C338	KM225263=KF917540	1961	*Aedes vigilax*	Australia
**Torres virus**	TS5273	KM225265	2000	Mosquito	Australia
**Spondweni virus**	Culex_quinquefasciatus/Haiti-1/2016	KX227369	1952	*Homo sapiens*	Nigeria
**Bagaza virus**	ARA23139_Dezidougou_CI_1988	MF380429	1988	*Culex poicilipes*	Cote dIvoire
**Israel turkey meningoencephalitis virus**	ME 30502	KF917537	1959	*Meleagris gallopavo*	Israel
**Rocio virus**	SPH 34675	NC_040776.1=MF461639.1=AY632542.4	1975	*Homo sapiens*	Brazil
**Ilheus virus**	BrMS-MQ10	KC481679	04/18/2010	*Aedes scapularis*	Brazil
**Ntaya virus**	Original	KF917539	1943	Mosquito	Uganda
**Tembusu virus**	BYD-1	JF312912	10/18/2010	Duck	China
	TMUV-JSGo	AB917090	12/01/2012	*Anser* sp.	China

	STWV	JX477686	2000	Broiler chicken	Malaysia
**Zika virus**	GD01	KU740184	02/2016	*Homo sapiens*	China
	ZIKV/Homo sapiens/NGA/IbH-30656_SM21V1-V3/1968	KU963574	09/09/1968	*Homo sapiens*	Nigeria
	Zika virus/Aedes taylori/Senegal/1984/DakAr41667	MF510857	06/12/1984	*Aedes taylori*	Senegal
**Sepik virus**	MK7148	NC_008719=DQ837642	1966	*Mansonia septempunctata*	Papua New Guinea
**Wesselsbron virus**	WSLV-IP262451/SEN/2014	KY056257	02/2014	*Homo sapiens*	Senegal
**Yellow fever virus**	Cahama-C17	KX982182	03/16/2016	*Homo sapiens*	Angola
	Uganda48a	AY968065	1948	*Homo sapiens*	Uganda
	Couma	DQ235229	1961	*Homo sapiens*	Ethiopia
	BeH413820	JF912181	1983	*Homo sapiens*	Brazil
	6A	KM388814	2005	*Homo sapiens*	Venezuela
	Ogbomosho	KU978763	1946	*Homo sapiens*	Nigeria
	Asibi	MF405338	1927	*Homo sapiens*	Ghana
**Entebbe bat virus**	UGA125	KP233893	06/2011	*Chaerephon pumila*	Niger
**Sokoluk virus**	LEIV-400K	NC_026624=KF917541	1970	*Vespertilio pipistrellus*	Kyrgyzstan
**Yokose virus**	XYBX1332	MH051229	07/2013	*Myotis daubentonii*	China
**Kedougou virus**	DakAar D1470	NC_012533=AY632540=DQ859061	1975?	Mosquito	Senegal
**Banzi virus**	SAH 336	DQ859056=NC_043110	1956	*Homo sapiens*	South Africa
**Bouboui virus**	DAK AR B490	NC_033693=DQ859057	1967	*Anopheles paludis*	Central African Republic
**Edge Hill virus**	YMP 48	NC_030289=DQ859060	2000	*Culex annulirostris*	Australia
**Jugra virus**	P-9-314	NC_033699=DQ859066	1969	*Aedes* spp	Malaysia
**Potiskum virus**	IBAN 10069	NC_029054=DQ859067	1987?	*Crycetomys gambianus*	Nigeria
**Saboya virus**	Dak AR D4600	NC_033697=DQ859062	1968	*Tatera kemp*	Senegal
**Uganda S virus**	Original	DQ859065	1971	*Aedes* spp.	Uganda
**dual host-affiliated Insect-Specific flaviviruses -dISFV- (n=13)**					
**Cháoyáng virus**	HLD115	JQ068102=NC_017086	Jun-2003	*Aedes vexans nipponii*	South Korea
**Lammi virus**	M0719	NC_024806	2007	Culicidae	Finland
	NA	FJ606789	2004	mosquito	Finland
**Nounané virus**	Nounane_B3	NC_033715=EU159426	2004	*Uranotaenia mashonaensis*	Cote d'Ivoire
**Barkedji virus**	Isolate 363/11	KC496020	2011	*Culex perexiguus*	Israel
	SQU29/Oman/2016	MG214905	2016	*Culex quinquefasciatus*	Oman

**Nhumirim virus**	BrMS-MQ10	NC_024017	2010	*Culex chidesteri*	Brazil
**Nanay virus**	PRD316/PER/09	MF139575	2009	*Melanoconion ocossa*	Peru
**Kampung Karu virus**	SWK_P44	KY320648	2013	*Anopheles tessellatus*	Malaysia
**Donggang virus**	DG0909	NC_016997	2009	*Aedes* sp.	China
**Marisma mosquito virus**	HU4528/07	MF139576	2003	*Ochlerotatus caspius*	Spain
**Ilomantsi virus**	M0724	NC_024805	2007	Mosquito	Finland
**Long Pine Key virus**	EVG 1-33	KY290249	2013	*Ochlerotatus atlanticus*	USA
**classical Insect-Specific flaviviruses -cISFV- (n=16)**					
**Aedes flavivirus**	Bangkok	KJ741266	2012	*Aedes albopictus*	Thailand
**cell fusing agent virus**	2	NC_001564=KJ741267	2012	*Aedes aegypti Galveston colony*	USA
**Culex flavivirus**	Otero 2009	KY349933	04/10/2013	*Culex quinquefasciatus*	Brazil
	CxFV_BR/ MT_CbaAr1256p2/2013	JQ518484	2006	*Culex pipiens*	China
	SDDM06-11	JX897904	2010	*Culex tritaeniorhynchus*	Taiwan
	TW100322	KX924632	07/17/2016	*Water flea*	USA
**Culex theileri flavivirus**	153	HE574573	2011	*Culex theileri*	Portugal
	JKT-8650	MF153378	01/21/1981	*Anopheles vagus*	Indonesia
**Hanko virus**	UNK	NC_030401=JQ268258	08/2005	Mosquito	Finland
**Kamiti River virus**	SR-75	AY149904	1999	*Aedes macintoshi*	Kenya
**mosquito flavivirus**	LSFlaviV-A20-09	NC_021069=KC464457	07/2009	*Culex tritaeniorhynchus*	China
	65_02	KX652377	08/03/2015	*Culex theileri*	Turkey
**Nakiwogo virus**	Uganda08	NC_030400	2008	*Mansonia africana nigerrima*	Uganda
**Nienokoue virus**	B51/CI/2004	NC_024299	2004	*Culex* sp	Cote d'Ivoire
**Palm creek virus**	56	NC_033694=KC505248	2010	*Coquillettidia xanthogaster*	Australia
**Quảng Bình virus**	DD1716	MG719525	08/2017	*Culex tritaeniorhynchus*	China
**Ecuador Paraiso Escondido virus**	Ecuador2012	NC_027999=KJ152564	2012	*Lutzomyia abonnenci*	Ecuador
**No Known Vector flaviviruses -NKVFV- (n=7)**					
**Apoi virus**	ApMAR	NC_003676=AF160193	NA	*Apodemu speciosus*	Japan
**Jutiapa virus**	JG-128	NC_026620=KJ469371	1969	*Sigmodon hispidus*	Guatemala
**Modoc virus**	M544	NC_003635=AJ242984	1956	*Peromyscus maniculatus*	USA
**Montana myotis leukoencephalitis virus**	Montana	NC_004119=AJ299445	1958	*Myotis lucifugus*	USA
**Batu Cave virus**	P70-1459	KJ469370	1971	*Cynopterus brachyotis*	Malaysia

**Phnom Penh bat virus**	30834_A38	NC_034007=KJ469372	1969	*Cynopterus brachyotis angulatus*	Cambodia
**Rio Bravo virus**	M64	JQ582840	1956	*Tadarida brasiliensis mexicana*	USA

Supplementary Table S3. Substitution saturation analysis.

Flavivirus group	I_{ss}[a]	$I_{ss.cSym}$[b]	$I_{ss.cAsym}$[c]	*p* value[d]	DF[e]
**NKVFV**	0.653	0.838	0.773	<0.0001	6692
**TBFV**	0.472	0.817	0.571	<0.0001	6581
**MBFV**	0.534	0.816	0.571	<0.0001	8129
**dISFV**	0.664	0.836	0.694	<0.0001	6991
**cISFV**	0.625	0.842	0.680	<0.0001	5799

[a]index of substitution saturation; [b]critical value for a symmetrical tree topology; [c]critical value for an asymmetrical tree topology; [d]probability that I_{ss} is significantly different from the critical values; [e]degrees of freedom

Supplementary Table S4. Substitution rates of viruses from the *Flaviviridae* family.

Numbers referring to Figure 2	Virus	Genus	Substitution rate (subs/site/year)	Date range (years)	Region	Reference
1	Pegivirus C (GBvirus C)	*Pegivirus*	$3.50x10^{-2}$	4	E1 coding region	(Simmonds et al. 2019; Romano et al. 2008)
2	Pegivirus C (GBvirus C)	*Pegivirus*	$1.65x10^{-2}$	4	NS5B coding region	(Simmonds et al. 2019; Romano et al. 2008)
3	Pegivirus C (GBvirus C)	*Pegivirus*	$9.33x10^{-3}$	3	E2 coding region	(Simmonds et al. 2019; Romano et al. 2008)
4	Pegivirus C (GBvirus C)	*Pegivirus*	$3.42x10^{-3}$	9	5’UTR	(Simmonds et al. 2019; Romano et al. 2008)
1	Hepatitis C virus	*Hepacivirus*	$2.10x10^{-3}$	17	E2-P7-NS2	(Simmonds et al. 2019; Magiorkinis et al. 2009)
2	Hepatitis C virus	*Hepacivirus*	$1.90x10^{-3}$	17	NSSB coding region	(Simmonds et al. 2019; Magiorkinis et al. 2009)
3	Hepatitis C virus	*Hepacivirus*	$1.42x10^{-3}$	19	Genome	(Simmonds et al. 2019; Gray et al. 2011)
4	Hepatitis C virus	*Hepacivirus*	$1.30x10^{-3}$	30	E2-P7-NS2	(Simmonds et al. 2019; Magiorkinis et al. 2009)
5	Hepatitis C virus	*Hepacivirus*	$1.20x10^{-3}$	17	NSSB coding region	(Simmonds et al. 2019; Magiorkinis et al. 2009)
6	Hepatitis C virus	*Hepacivirus*	$1.17x10^{-3}$	19	Genome	(Simmonds et al. 2019; Gray et al. 2011)
7	Hepatitis C virus	*Hepacivirus*	$1.12x10^{-3}$	19	Genome	(Simmonds et al. 2019; Gray et al. 2011)
8	Hepatitis C virus	*Hepacivirus*	$1.00x10^{-3}$	30	NSSB with E2P7-NS2 rate prior	(Simmonds et al. 2019; Magiorkinis et al. 2009)
9	Hepatitis C virus	*Hepacivirus*	$1.00x10^{-3}$	30	NSSB coding region	(Simmonds et al. 2019; Magiorkinis et al. 2009)

1	Dengue virus type 4	*Flavivirus*	1.07×10^{-3}	26	E coding region	(Simmonds et al. 2019; Klungthong et al. 2004)
2	Dengue virus type 3	*Flavivirus*	1.03×10^{-3}	18	E coding region	(Simmonds et al. 2019; Fajardo et al. 2009)
3	Zika virus	*Flavivirus*	1.00×10^{-3}	49	Genome	(Faria et al. 2016)
4	Dengue virus type 3	*Flavivirus*	9.01×10^{-4}	44	E coding region	(Simmonds et al. 2019; Twiddy et al. 2003)
5	Dengue virus type 3	*Flavivirus*	8.90×10^{-4}	50	E coding region	(Simmonds et al. 2019; Araújo et al. 2009)
6	Dengue virus type 3	*Flavivirus*	8.48×10^{-4}	7	E coding region	(Simmonds et al. 2019; Ramírez et al. 2010)
7	West Nile virus	*Flavivirus*	8.50×10^{-4}	8	E coding region	(Simmonds et al. 2019; Bertolotti et al. 2007)
8	Dengue virus type 4	*Flavivirus*	8.30×10^{-4}	18	E coding region	(Simmonds et al. 2019; Carrington et al. 2005)
9	Dengue virus type 2	*Flavivirus*	8.00×10^{-4}	21	E coding region	(Simmonds et al. 2019; Carrington et al. 2005)
10	Dengue virus type 1	*Flavivirus*	6.50×10^{-4}	43	E coding region	(Simmonds et al. 2019; Patil et al. 2011)
11	Dengue virus type 2	*Flavivirus*	6.50×10^{-4}	51	E coding region	(Simmonds et al. 2019; Kumar et al. 2010)
12	Dengue virus type 2	*Flavivirus*	6.07×10^{-4}	54	E coding region	(Simmonds et al. 2019; Twiddy et al. 2003)
13	Dengue virus type 4	*Flavivirus*	6.02×10^{-4}	38	E coding region	(Simmonds et al. 2019; Twiddy et al. 2003)
14	Dengue virus type 2	*Flavivirus*	5.66×10^{-4}	19	E coding region	(Simmonds et al. 2019; Foster et al. 2004)
15	Dengue virus type 1	*Flavivirus*	4.55×10^{-4}	54	E coding region	(Simmonds et al. 2019; Twiddy et al. 2003)
16	Japanese encephalitis virus	*Flavivirus*	4.35×10^{-4}	74	Genome	(Simmonds et al. 2019; Mohammed et al. 2011)
17	Yellow fever virus	*Flavivirus*	4.20×10^{-4}	76	prM/E	(Simmonds et al. 2019; Bryant et al. 2007)
18	St.Louis encephalitis virus	*Flavivirus*	4.10×10^{-4}	72	E coding region	(Simmonds et al. 2019; Auguste et al. 2009)
19	West Nile virus lineage 2	*Flavivirus*	2.73×10^{-4}	74	Genome	(McMullen et al. 2013)
20	West Nile virus USA	*Flavivirus*	2.70×10^{-4}	14	Genome	(Di Giallonardo et al. 2015)
21	St.Louis encephalitis virus	*Flavivirus*	2.17×10^{-4}	78	E coding region	(Simmonds et al. 2019; Baillie et al. 2008)
1	Kyasanur forest disease virus	*Flavivirus*	6.40×10^{-4}	49	Envelop-NS5	(Simmonds et al. 2019; Mehla et al. 2009)
2	Tick-borne encephalitis virus	*Flavivirus*	1.40×10^{-4}	75	E genome region	(Subbotina and Loktev 2012)
3	Omsk haemorrhagic fever virus	*Flavivirus*	1.38×10^{-4}	60	E coding region	(Karan et al. 2014)
4	Powassan virus	*Flavivirus*	5.40×10^{-5}	54	NS5 genome region	(Subbotina and Loktev 2012)
5	Tick-borne encephalitis virus	*Flavivirus*	3.30×10^{-5}	59	Genome	(Clark et al. 2020)

6	Louping ill virus	*Flavivirus*	$1.90x10^{-5}$	84	Genome	(Clark et al. 2020)
1	Culex flavivirus	*Flavivirus*	$1.09x10^{-3}$	15	E coding region	This work
2	Cell-fusing agent virus	*Flavivirus*	$5.01x10^{-5}$	41	E coding region	This work

Supplementary Table S5. Single-likelihood ancestor counting (SLAC) for all flavivirus viral ORFs.

**ORF**	**SLAC (dN/dS)**				
	**NKVFV**	**TBFV**	**MBFV**	**dISFV**	**cISFV**
**C**	0.0937	0.195	0.149	0.141	0.127
**E**	0.108	0.0891	0.085	0.0905	0.108
**prM**	0.151	0.129	0.0745	0.089	0.1
**ns1**	0.113	0.0945	0.0912	0.0866	0.0985
**ns2a**	0.15	0.15	0.115	0.071	0.168
**ns2b**	0.147	0.106	0.0892	0.106	0.199
**ns3**	0.0838	0.0743	0.0612	0.0697	0.092
**ns4a**	0.177	0.099	0.0838	0.0857	0.0964
**ns4b**	0.144	0.0932	0.0713	0.078	0.125
**ns5**	0.0921	0.0739	0.0698	0.0595	0.0717

Supplementary Table S6. Likelihood ratio test statistics for branch site tests.

	**BUSTED**		**MAvsMA1**		**N° of sites[b]**	**RELAX[c]**	
**Flavivirus Group/Node**	**- ΔlnL[a]**	***p* value (fdr correction)**	**- ΔlnL[a]**	***p* value (fdr correction)**		**k**	***p* value**
**TBFV**							
**Node 26**	2.45	0.118 (0.118)	65.10	$7.13x10^{-16}$ ($7.13x10^{-16}$)	NA	NA	NA
**Node 29**	31.49	$2.00x10^{-08}$ ($3.00x10^{-08}$)	103.50	$2.60x10^{-24}$ ($7.81x10^{-24}$)	62	1.11	0.185
**Node 31**	42.78	$6.11x10^{-11}$ ($1.83x10^{-10}$)	98.32	$3.56x10^{-23}$ ($5.34x10^{-23}$)	74	0.85	0.173
**MBFV**							
**Node 5**	16.85	$4.05x10^{-05}$ ($4.87x10^{-05}$)	16.36	$5.2x10^{-05}$ ($5.96x10^{-05}$)	NA	0.82	0.007
**Node 7**	25.14	$5.33x10^{-07}$ ($9.00x10^{-07}$)	162.99	$2.52x10^{-37}$ ($1.51x10^{-36}$)	NA	0.97	0.9
**Node 9**	30.43	$3.47x10^{-08}$ ($9.10x10^{-08}$)	75.96	$2.89x10^{-18}$ ($9.33x10^{-18}$)	NA	1.55	0.003
**Node 10**	21.58	$3.39x10^{-06}$ ($4.91x10^{-06}$)	234.68	$5.69x10^{-53}$ ($5.97x10^{-52}$)	NA	1.61	0.009

**Node 11**	19.91	$8.13x10^{-06}$ ($1.07x10^{-05}$)	34.46	$4.35x10^{-09}$ ($7.61x10^{-09}$)	1	1.01	0.619
**Node 12**	17.25	$3.28x10^{-05}$ ($4.06x10^{-05}$)	166.4194	$4.48x10^{-38}$ ($3.14x10^{-37}$)	NA	1.41	0.001
**Node 13**	21.82	$2.99x10^{-06}$ ($4.48x10^{-06}$)	81.79	$1.51x10^{-19}$ ($5.30x10^{-19}$)	NA	2.08	0.007
**Node 17**	2.64	0.10 ($1.10x10^{-01}$)	8.81	0.003 ($3.23x10^{-03}$)	NA	NA	NA
**Node 30**	16.22	$5.65x10^{-05}$ ($6.59x10^{-05}$)	285.95	$3.80x10^{-64}$ ($7.98x10^{-63}$)	NA	1.63	0.001
**Node 32**	20.58	$5.72x10^{-06}$ ($8.01x10^{-06}$)	33.35	$7.68x10^{-09}$ ($1.29x10^{-08}$)	NA	1.05	<0.001
**Node 33**	23.86	$1.03x10^{-06}$ ($1.67x10^{-06}$)	110.77	$6.65x10^{-26}$ ($2.79x10^{-25}$)	NA	2.09	<0.001
**Node 35**	26.99	$2.04x10^{-07}$ ($3.90x10^{-07}$)	48.32	$3.61x10^{-12}$ ($8.43x10^{-12}$)	2	1.62	<0.001
**Node 38**	23.65	$1.16x10^{-06}$ ($1.80x10^{-06}$)	38.22	$6.32x10^{-10}$ ($1.21x10^{-09}$)	5	1.17	0.167
**Node 43**	7.78	$5.27x10^{-03}$ ($5.68x10^{-03}$)	21.42	$3.68x10^{-06}$ ($4.68x10^{-06}$)	1	0.98	0.001
**Node 48**	20.49	$6.00x10^{-06}$ ($8.13x10^{-06}$)	28.47	$9.51x10^{-08}$ ($1.54x10^{-07}$)	1	1.11	0.036
**Node 49**	31.25	$2.27x10^{-08}$ ($6.35x10^{-08}$)	223.24	$1.77x10^{-50}$ ($1.49x10^{-49}$)	NA	1.39	0.068
**Node 50**	38.28	$6.12x10^{-10}$ ($6.42x10^{-09}$)	23.89	$1.02x10^{-06}$ ($1.38x10^{-06}$)	4 (one in common with nodes 109 and 120)	0.94	0.955
**Node 81**	29.59	$5.37x10^{-08}$ ($1.25x10^{-07}$)	39.48	$3.31x10^{-10}$ ($6.62x10^{-10}$)	7	1.24	0.083
**Node 96**	33.81	$6.08x10^{-09}$ ($2.32x10^{-08}$)	24	$9.65x10^{-07}$ ($1.35x10^{-06}$)	2	0.59	<0.001
**Node 105**	36.01	$1.96x10^{-09}$ ($1.17x10^{-08}$)	96.4	$9.36x10^{-23}$ ($3.57x10^{-22}$)	NA	1.66	<0.001
**Node 106**	37.55	$8.93x10^{-10}$ ($7.50x10^{-09}$)	379.52	$1.58x10^{-84}$ ($6.63x10^{-83}$)	NA	1.11	0.058
**Node 108**	32.13	$1.44x10^{-08}$ ($4.66x10^{-08}$)	251.36	$1.31x10^{-56}$ ($1.84x10^{-55}$)	3	1.32	0.057
**Node 109**	19.59	$9.57x10^{-06}$ ($1.22x10^{-05}$)	36.58	$1.47x10^{-09}$ ($2.68x10^{-09}$)	5 (one in common with nodes 50 and 120)	1.14	0.343
**Node 115**	37.06	$1.15x10^{-09}$ ($8.02x10^{-09}$)	62.15	$3.17x10^{-15}$ ($8.89x10^{-15}$)	NA	1.77	<0.001
**Node 116**	29.62	$5.25x10^{-08}$ ($1.25x10^{-07}$)	0.04	0.84 ($8.48x10^{-01}$)	NA	NA	NA
**Node 117**	25.13	$5.36x10^{-07}$ ($9.00x10^{-07}$)	26.12	$3.20x10^{-07}$ ($4.81x10^{-07}$)	1	1.01	0.505
**Node 120**	27.08	$1.95x10^{-07}$ ($3.90x10^{-07}$)	18.43	$1.76x10^{-05}$ ($2.11x10^{-05}$)	2 (one in common with nodes 50 and 109)	1.02	0.453
**Node 123**	42.7	$6.39x10^{-11}$ ($1.34x10^{-09}$)	25.85	$3.69x10^{-07}$ ($5.35x10^{-07}$)	1	1.10	0.097
**Node 124**	26.35	$2.84x10^{-07}$ ($5.20x10^{-07}$)	22.68	$1.91x10^{-06}$ ($2.51x10^{-06}$)	NA	1.12	0.422

**Node 126**	28.1	$1.16x10^{-07}$ ($2.43x10^{-07}$)	151.98	$6.40x10^{-35}$ ($2.99x10^{-34}$)	5	1.13	0.288
**Node 127**	34.93	$3.42x10^{-09}$ ($1.79x10^{-08}$)	66.64	$3.25x10^{-16}$ ($9.75x10^{-16}$)	NA	1.19	0.351
**Node 129**	34.1	$5.23x10^{-09}$ ($2.20x10^{-08}$)	27.93	$1.26x10^{-07}$ ($1.96x10^{-07}$)	10	1.05	0.696
**Node 148**	150.59	$1.29x10^{-34}$ ($5.42x10^{-33}$)	161.16	$6.31x10^{-37}$ ($3.31x10^{-36}$)	2	1.28	0.018
**Node 149**	9.78	$1.77x10^{-03}$ ($1.95x10^{-03}$)	46.27	$1.03x10^{-11}$ ($2.28x10^{-11}$)	NA	1.04	0.891
**Node 159**	0.54	0.46 (0.47)	7.49	$6.20x10^{-03}$ ($6.35x10^{-03}$)	NA	NA	NA
**Node 168**	15.3	$9.19x10^{-05}$ ($1.04x10^{-04}$)	17.25	$3.27x10^{-05}$ ($3.82x10^{-05}$)	3	0.90	0.111
**Node 175**	38.82	$4.65x10^{-10}$ ($6.42x10^{-09}$)	57.1	$4.14x10^{-14}$ ($1.09x10^{-13}$)	NA	1.78	<0.001
**Node 176**	34.34	$4.62x10^{-09}$ ($2.15x10^{-08}$)	56.1	$6.90x10^{-14}$ ($1.70x10^{-13}$)	NA	1.77	<0.001
**Node 177**	31.75	$1.75x10^{-08}$ ($5.26x10^{-08}$)	18.67	$1.55x10^{-05}$ ($1.91x10^{-05}$)	1	0.89	0.325
**Node 180**	33.49	$7.15x10^{-09}$ ($2.50x10^{-08}$)	7.76	$5.33x10^{-03}$ ($5.59x10^{-03}$)	1	1.00	1
**Node 186**	28.3	$1.04x10^{-07}$ ($2.29x10^{-07}$)	39.96	$2.58x10^{-10}$ ($5.43x10^{-10}$)	5	1.28	0.034
**Node 191**	0.15	0.70 ($6.99x10^{-01}$)	10.93	$9.47x10^{-04}$ ($1.05x10^{-03}$)	NA	NA	NA
**NKVFV**							
**Node4**	0.46	0.50 (0.51)	132.36	$1.29x10^{-30}$ ($2.50x10^{-30}$)	NA	NA	NA
**Node7**	0.43	0.51 (0.51)	1.24	0.26 (0.26)	NA	NA	NA

[a] 2ΔlnL is twice the difference of the natural logs of the maximum likelihood of the models being compared.
[b] To identified sites evolving under positive selection on specific branches, the BEB analysis from MA (with a cutoff of 0.95) was used
[c] RELAX method was applied only for nodes identified under episodic positive selection by aBSREL and confirmed by both MAvsMA1 and BUSTED

References

Araújo JM, Nogueira RM, Schatzmayr HG, Zanotto PM, Bello G. Phylogeography and evolutionary history of dengue virus type 3. Infect Genet Evol. 2009;9(4):716-725.

Auguste AJ, Pybus OG, Carrington CV. Evolution and dispersal of St. Louis encephalitis virus in the Americas. Infect Genet Evol. 2009;9(4):709-715.

Baillie GJ, Kolokotronis SO, Waltari E, Maffei JG, Kramer LD, Perkins SL. Phylogenetic and evolutionary analyses of St. Louis encephalitis virus genomes. Mol Phylogenet Evol. 2008;47(2):717-728.

Bertolotti L, Kitron U, Goldberg TL. Diversity and evolution of West Nile virus in Illinois and the United States, 2002-2005. Virology. 2007;360(1):143-149.

Bryant JE, Holmes EC, Barrett AD. Out of Africa: a molecular perspective on the introduction of yellow fever virus into the Americas. PLoS Pathog. 2007;3(5):e75.

Carrington CV, Foster JE, Pybus OG, Bennett SN, Holmes EC. Invasion and maintenance of dengue virus type 2 and type 4 in the Americas. J Virol. 2005;79(23):14680-14687.

Clark JJ, Gilray J, Orton RJ, Baird M, Wilkie G, Filipe ADS, Johnson N, McInnes CJ, Kohl A, Biek R. Population genomics of louping ill virus provide new insights into the evolution of tick-borne flaviviruses. PLoS Negl Trop Dis. 2020;14(9):e0008133.

Di Giallonardo F, Geoghegan JL, Docherty DE, McLean RG, Zody MC, Qu J, Yang X, Birren BW, Malboeuf CM, Newman RM, Ip HS, Holmes EC. Fluid Spatial Dynamics of West Nile Virus in the United States: Rapid Spread in a Permissive Host Environment. J Virol. 2015;90(2):862-872.

Fajardo A, Recarey R, de Mora D, D'Andrea L, Alvarez M, Regato M, Colina R, Khan B, Cristina J. Modeling gene sequence changes over time in type 3 dengue viruses from Ecuador. Virus Res. 2009;141(1):105-109.

Faria NR, Azevedo RD, Kraemer MU, Souza R, Cunha MS, Hill SC, Theze J, Bonsall MB, Bowden TA, Rissanen I, Rocco IM, Nogueira JS, Maeda AY, Vasami FG, Macedo FL, Suzuki A, Rodrigues SG, Cruz AC, Nunes BT, Medeiros DB, Rodrigues DS, Nunes Queiroz AL, Silva EV, Henriques DF, Travassos da Rosa ES, de Oliveira CS, Martins LC, Vasconcelos HB, Casseb LM, Simith DB, Messina JP, Abade L, Lourenco J, Alcantara LC, Lima MM, Giovanetti M, Hay SI, de Oliveira RS, Lemos PD, Oliveira LF, de Lima CP, da Silva SP, Vasconcelos JM, Franco L, Cardoso JF, Vianez-Junior JL, Mir D, Bello G, Delatorre E, Khan K, Creatore M, Coelho GE, de Oliveira WK, Tesh R, Pybus OG, Nunes MR, Vasconcelos PF. Zika virus in the Americas: Early epidemiological and genetic findings. Science. 2016; 352(6283):345-349.

Foster JE, Bennett SN, Carrington CV, Vaughan H, McMillan WO. Phylogeography and molecular evolution of dengue 2 in the Caribbean basin, 1981-2000. Virology. 2004;324(1):48-59.

Gray RR, Parker J, Lemey P, Salemi M, Katzourakis A, Pybus OG. The mode and tempo of hepatitis C virus evolution within and among hosts. BMC Evol Biol. 2011;11:131-2148-11-131.

Karan LS, Ciccozzi M, Yakimenko VV, Lo Presti A, Cella E, Zehender G, Rezza G, Platonov AE. The deduced evolution history of Omsk hemorrhagic fever virus. J Med Virol. 2014;86(7):1181-1187.

Klungthong C, Zhang C, Mammen MP,Jr, Ubol S, Holmes EC. The molecular epidemiology of dengue virus serotype 4 in Bangkok, Thailand. Virology. 2004;329(1):168-179.

Kumar SR, Patil JA, Cecilia D, Cherian SS, Barde PV, Walimbe AM, Yadav PD, Yergolkar PN, Shah PS, Padbidri VS, Mishra AC, Mourya DT. Evolution, dispersal and replacement of American genotype

dengue type 2 viruses in India (1956-2005): selection pressure and molecular clock analyses. J Gen Virol. 2010;91(Pt 3):707-720.

Magiorkinis G, Magiorkinis E, Paraskevis D, Ho SY, Shapiro B, Pybus OG, Allain JP, Hatzakis A. The global spread of hepatitis C virus 1a and 1b: a phylodynamic and phylogeographic analysis. PLoS Med. 2009;6(12):e1000198.

McMullen AR, Albayrak H, May FJ, Davis CT, Beasley DWC, Barrett ADT. Molecular evolution of lineage 2 West Nile virus. J Gen Virol. 2013;94(Pt 2):318-325.

Mehla R, Kumar SR, Yadav P, Barde PV, Yergolkar PN, Erickson BR, Carroll SA, Mishra AC, Nichol ST, Mourya DT. Recent ancestry of Kyasanur Forest disease virus. Emerg Infect Dis. 2009;15(9):1431-1437.

Mohammed MA, Galbraith SE, Radford AD, Dove W, Takasaki T, Kurane I, Solomon T. Molecular phylogenetic and evolutionary analyses of Muar strain of Japanese encephalitis virus reveal it is the missing fifth genotype. Infect Genet Evol. 2011;11(5):855-862.

Patil JA, Cherian S, Walimbe AM, Patil BR, Sathe PS, Shah PS, Cecilia D. Evolutionary dynamics of the American African genotype of dengue type 1 virus in India (1962-2005). Infect Genet Evol. 2011;11(6):1443-1448.

Ramírez A, Fajardo A, Moros Z, Gerder M, Caraballo G, Camacho D, Comach G, Alarcón V, Zambrano J, Hernández R, Moratorio G, Cristina J, Liprandi F. Evolution of dengue virus type 3

genotype III in Venezuela: diversification, rates and population dynamics. Virol J. 2010;7:329-422X-7-329.

Romano CM, Zanotto PM, Holmes EC. Bayesian coalescent analysis reveals a high rate of molecular evolution in GB virus C. J Mol Evol. 2008;66(3):292-297.

Simmonds P, Aiewsakun P, Katzourakis A. Prisoners of war - host adaptation and its constraints on virus evolution. Nat Rev Microbiol. 2019;17(5):321-328.

Subbotina EL, Loktev VB. Molecular evolution of the tick-borne encephalitis and Powassan viruses. Mol Biol (Mosk). 2012;46(1):82-92.

Twiddy SS, Holmes EC, Rambaut A. Inferring the rate and time-scale of dengue virus evolution. Mol Biol Evol. 2003;20(1):122-129.

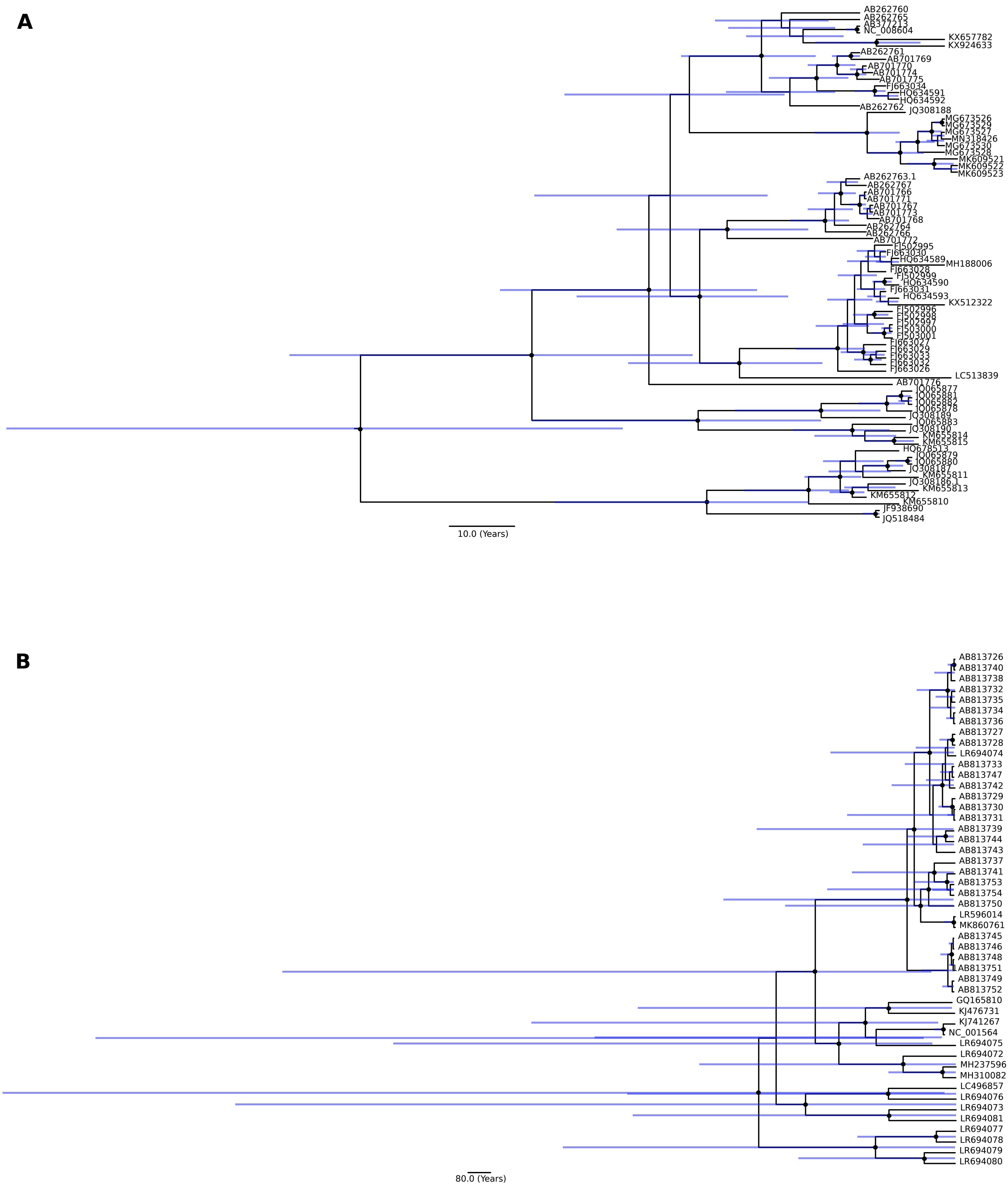

Supplementary Figure S1. cISFVs tree. A time-calibrated maximum clade credibility tree generated using TreeAnnotator for (**A**) Culex flavivirus (CxFV) and (**B**) Cell fusing agent virus (CFAV) envelope proteins. For internal nodes 95% credible interval bars are shown and black dots indicate a posterior probability > 0.80 for that node.

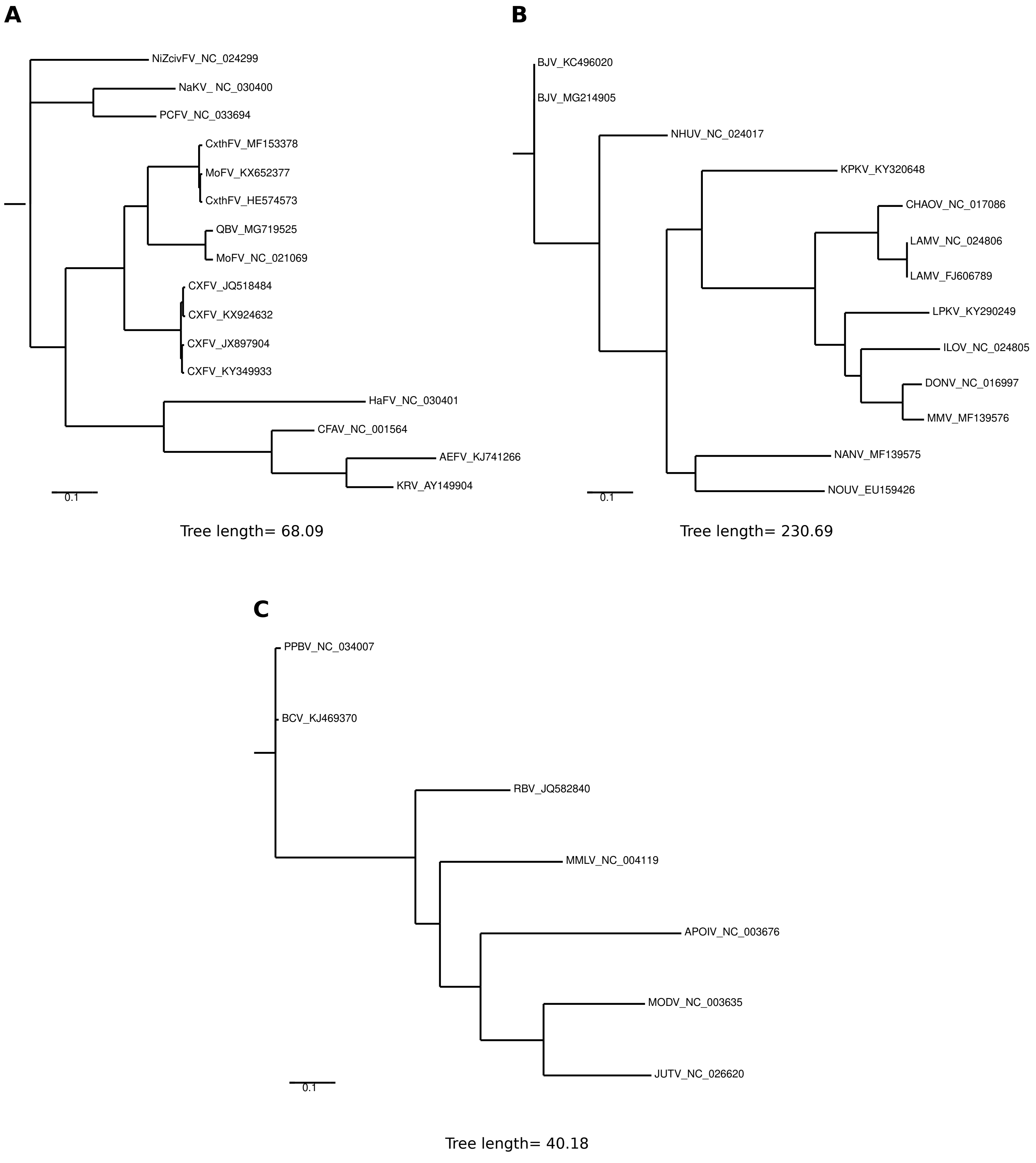

Supplementary Figure S2. Phylogenetic trees. Maximum likelihood phylogeny tree for (**A**) cISFVs (**B**) dISFVs and (**C**) NKVFVs.
